# Integrative Inflammation–Metabolism Indicator for Cardiovascular–Kidney–Metabolic Syndrome: Evaluating the C‐Reactive Protein–Triglyceride Glucose Index for Risk Stratification and Progression Across Three National Cohorts

**DOI:** 10.1155/mi/8912366

**Published:** 2026-07-28

**Authors:** Yupeng Zeng, Huhao Feng, Haiying Cheng, Weiqing Wu, Hui He, Lixin Cheng, Qingshan Geng

**Affiliations:** ^1^ The Second Clinical Medical College, Jinan University, Shenzhen 518020, China, jnu.edu.cn; ^2^ Shenzhen People’s Hospital (The Second Clinical Medical College, Jinan University; The First Affiliated Hospital, Southern University of Science and Technology), Shenzhen 518020, China, szhospital.com; ^3^ School of Medicine, Southern University of Science and Technology, Shenzhen 518055, China, sustc.edu.cn; ^4^ Department of Health Management, Shenzhen People’s Hospital (The Second Clinical Medical College, Jinan University; The First Affiliated Hospital, Southern University of Science and Technology), Shenzhen 518020, China, szhospital.com

**Keywords:** C-reactive protein–triglyceride glucose index, cardiovascular–kidney–metabolic syndrome, inflammation, insulin resistance, machine learning, multicenter cohort study

## Abstract

**Background:**

Inflammation plays a critical role in the onset and progression of cardiovascular–kidney–metabolic (CKM) syndrome. However, the optimal inflammatory biomarker that simultaneously reflects disease severity and predicts future progression of CKM remains unexplored.

**Methods:**

This study utilized data from three nationally representative cohorts: National Health and Nutrition Examination Survey (NHANES), UK Biobank (UKB), and China Health and Retirement Longitudinal Study (CHARLS). In NHANES, three machine learning approaches were applied to identify the inflammatory biomarker most strongly associated with CKM stages. The association between this biomarker and CKM severity was validated in UKB and CHARLS. Its predictive value for CKM progression was examined in UKB and CHARLS with Cox proportional hazard models. Multiple sensitivity analyses were conducted to ensure the robustness of the findings.

**Results:**

Among 15 circulating inflammatory biomarkers, the C‐reactive protein–triglyceride glucose index (CTI) was identified as the most informative indicator of advanced CKM risk. In the cross‐sectional analyses, 8959 participants from NHANES, 208,625 from UKB, and 8550 from CHARLS were included. After adjustment for potential confounders, higher CTI levels were consistently associated with advanced CKM across the three cohorts (NHANES: odds ratio [OR] = 1.35, 95% confidence interval (CI): 1.20–1.48; UKB: OR = 1.55, 95% CI: 1.46–1.64; CHARLS: OR = 1.42, 95% CI: 1.28–1.60). In longitudinal analyses including 186,753 participants from UKB and 3673 from CHARLS, elevated CTI levels predicted an increased risk of incident advanced CKM (UKB: HR = 1.22, 95% CI: 1.17–1.30; CHARLS: HR = 1.21, 95% CI: 1.01–1.46).

**Conclusions:**

CTI emerged as the most robust inflammatory biomarker for assessing disease severity and predicting CKM progression among multiple candidates. Its reproducible associations across national cohorts support its utility for early detection and risk stratification, offering a basis to refine CKM management.

## 1. Introduction

Substantial global morbidity and mortality are attributed to cardiovascular disease (CVD), chronic kidney disease (CKD), and metabolic disorders [1]. Accumulating clinical and epidemiological evidence reveals a close interplay between these disorders [2–4]. Cardiovascular–kidney–metabolic (CKM) syndrome, recently introduced by the American Heart Association (AHA) in 2023, is a systemic disorder resulting from complex interactions among metabolic risk factors, CKD, and CVD [5]. According to the Global Burden of Disease Study 2021, CKM syndrome and its key components constitute a leading and growing global public health burden across all sociodemographic regions and income settings.

CKM is classified into five stages (stages 0–4) according to the presence of risk factors and established diseases, with stages 3 and 4 defined as advanced CKM requiring more comprehensive management [5–7]. Inflammation is recognized as a core pathogenic mechanism linking metabolic dysregulation, CKD, and CVD. Although inflammatory markers have been associated with individual CKM components, the evidence regarding an optimal inflammatory marker that simultaneously reflects CKM severity and predicts progressive disease remains limited.

Indicators from blood testing, owing to their convenience, are commonly used to assess an individual’s inflammatory status, such as C‐reactive protein (CRP) and white blood cell count (WBC). Recently, composite inflammatory indices—including the CRP‐triglyceride glucose index (CTI) and neutrophil percentage‐to‐albumin ratio (NPAR)—have emerged as more sensitive tools for integrating inflammatory and metabolic status [8, 9]. While individual studies have linked these markers to CKM [10–12], no systematic comparison of multiple inflammatory biomarkers has been performed, and the added value of composite indices remains unclear.

To address these research gaps, we analyzed three nationally representative cohorts: the UK Biobank (UKB), the United States National Health and Nutrition Examination Survey (NHANES), and the China Health and Retirement Longitudinal Study (CHARLS). Cross‐population validation strengthens generalizability and reduces population‐specific bias, which is critical for global applicability. Machine learning methods were used to enable systematic, unbiased feature selection, overcoming the limitations of traditional univariate comparisons. This study specifically aimed to (1) identify the most informative inflammatory biomarker for advanced CKM using machine learning and conventional statistical approaches, (2) evaluate the cross‐sectional association between the selected biomarker and CKM severity, and (3) assess its longitudinal association with incident advanced CKM to strengthen the robustness of the findings.

## 2. Methods

### 2.1. Data Sources and Study Population

The UKB, NHANES, and CHARLS were all nationally representative cohorts conducted in the United Kingdom, United States, and China, respectively [6, 13, 14]. Detailed information of the three cohorts is presented in Supporting Information 1. For the cross‐sectional study, data from the UKB recruitment stage (2006–2010), NHANES (1999–2009), and wave 1 of CHARLS (2011) were included. All the individuals of the three cohorts with missing data of inflammation biomarkers and CKM indicators were excluded, remaining 8959 participants from NHANES, 208,625 from UKB, and 8550 from CHARLS. While for the longitudinal study, data from UKB and CHARLS (wave1–wave3) were included. After excluding people identified as advanced CKM at the baseline and with missing information for follow‐up, 186,753 individuals from UKB and 3673 from CHARLS remained, respectively. The flowchart of the study design and details of the participant selection procedure are shown in Supporting Information 2: Figures S1–S4.

The NHANES, UKB, and CHARLS were approved by the National Center for Health Statistics Research Ethics Review Board, North West Multicenter Research Ethics Committee, and Ethics Review Committees of Peking University. Informed consent was obtained from each participant in these three cohorts.

### 2.2. Ascertainment for Inflammation Biomarkers

We included 15 inflammation biomarkers derived from blood samples. All the calculation formulas were adopted from the previous literature [10, 15–17]. The formulas are as follows: CRP, CTI = 0.412 × Ln (CRP [mg/L]) + Ln (TG [mg/dL] × FPG [mg/dL])/2, NPAR = neutrophil percentage/albumin (g/mL), neutrophil‐to‐lymphocyte ratio (NLR) = neutrophil count/lymphocyte count, neutrophil‐to‐monocyte ratio (NMR) = neutrophil count/monocyte count, platelet‐to‐lymphocyte ratio (PLR) = platelet count/‐lymphocyte count, platelet‐to‐neutrophil ratio (PNR) = platelet count/neutrophil count, platelet‐to‐monocyte ratio (PMR) = platelet count/monocyte count, monocyte‐to‐lymphocyte ratio (MLR) = monocyte count/lymphocyte count, systemic immune inflammation index (SII) = platelet count × neutrophil count/lymphocyte count, aggregate index of systemic inflammation (AISI) = neutrophil count × platelet count × monocyte count/lymphocyte count, CRP‐to‐albumin ratio (CAR) = CRP/albumin, neutrophil to high‐density lipoprotein cholesterol ratio (NHR) = neutrophil count/HDL‐C, leukocyte to high‐density lipoprotein cholesterol ratio (LHR) = leukocyte count/HDL‐C, and monocyte to high‐density lipoprotein cholesterol ratio (MHR) = monocyte count/HDL‐C. Due to unit and dimensionality considerations, the following indices—AISI, SII, PLR, PNR, and PMR—have been logarithmically transformed and are designated as AISI‐log, SII‐log, PLR‐log, PNR‐log, and PMR‐log.

### 2.3. Definition for CKM Syndrome

CKM syndrome characterizes the complex interplay between CVD, CKD, and metabolic dysregulation. CKM stages (0–4) were classified according to the AHA Presidential Advisory framework, which delineates a spectrum of escalating cardiometabolic and renal risk [5]. Stage 0: Denotes the absence of CKM risk factors. Stage 1: Defined by the presence of excess or dysfunctional adiposity. Stage 2: Characterized by established metabolic risk factors and/or CKD. Stage 3: Included individuals without diagnosed clinical CVD but possessing either a very‐high‐risk CKD or a high predicted 10‐year CVD risk. The 10‐year CVD risk was assessed using the AHA Predicting Risk of CVD EVENTS (PREVENT) equations [18, 19]. CKD risk stratification was performed according to the criteria recommended by the Improving Global Kidney Disease Prognosis Organization (KDIGO) [5]. Stage 4: Encompassed individuals with existing clinical CVD. The detailed descriptions of CKM syndrome stage definitions and related medical conditions are shown in Supporting Information 1 and Supporting Information 3: Table S1. Additionally, advanced CKM syndrome was defined as stages 3 and 4 of CKM syndrome, and CKM syndrome 0–2 stages were defined as nonadvanced CKM syndrome [6, 7].

### 2.4. Assessments of Covariates

Covariate selection was conducted on the basis of the epidemiological literature and clinical pathological relevance [20, 21]. To ensure consistent adjustment and maximize cross‐cohort comparability, only covariates uniformly available across all three cohorts were included. These covariates included sociodemographic data (age, gender, and education levels), lifestyle factors (smoking status and drinking status), physical measurements (body mass index [BMI], systolic blood pressure [SBP], and diastolic blood pressure [DBP]), laboratory indicators (low‐density lipoprotein cholesterol [LDL‐C] and estimated glomerular filtration rate [eGFR]), medication history (antidiabetic drugs and lipid‐lowering drugs), and baseline CKM stage (categorized as stages 0, 1, and 2). Detailed grouping settings and reference groups for categorical variables are provided in Supporting Information 1.

### 2.5. Screening for the Optimal Inflammation Biomarker

To identify candidate biomarkers associated with prevalent advanced CKM, feature selection was performed in the NHANES dataset as a discovery cohort, given its multiethnic composition and diverse metabolic and inflammatory profiles. The selected biomarker was subsequently validated in independent cohorts, including UKB and CHARLS. We conducted 3 ML methods to screen important inflammatory biomarkers, including Least Absolute Shrinkage and Selection Operator (LASSO), Boruta algorithm, and support vector machine–recursive feature elimination (SVM‐RFE). These algorithms were chosen for their complementary strengths in feature selection, collectively enhancing the robustness and accuracy of identifying inflammatory biomarkers [22]. Details of these machine learning algorithms are presented in Supporting Information 1. The overlapping inflammatory biomarkers identified by the 3 ML algorithms were selected as key markers for advanced CKM. We further utilized receiver operating characteristic (ROC) curves and performed correlation analyses. Finally, the most important and strongly associated biomarker was selected for subsequent analyses based on the above screening procedures.

### 2.6. Statistical Analyses

All the characteristics were presented as mean (SD) for continuous variables and as frequencies and proportions for categorical variables. All continuous covariates were directly included in the regression analysis. For the cross‐sectional analyses, data from all three cohorts were included. Participants were divided into three groups based on an equal‐frequency tertile cutoff of CTI values within each individual cohort, with the T1 group as the reference. Three distinct regression models were constructed: Model 1, crude unadjusted model; Model 2, adjusted for age and gender; and Model 3, further adjusted for education, smoking status, drinking status, BMI, SBP, DBP, LDL‐C, eGFR, antidiabetic drugs, lipid‐lowering drugs, and baseline CKM stage (stages 0, 1, and 2). In addition, fully adjusted restricted cubic spline (RCS) analyses were utilized to explore the potential nonlinear association between the CTI and the risk of advanced CKM. While for the longitudinal analyses, data from UKB and CHARLS were included. After excluding participants with advanced CKM in the baseline, the Kaplan–Meier curve was used to estimate the cumulative risk of new‐onset advanced CKM based on different CTI tertiles, with differences assessed by the log‐rank test. Three Cox proportional hazard regression models were constructed, with adjustments for confounders consistent with the cross‐sectional study. Schoenfeld residual test was adopted to verify the Cox proportional hazards assumption, and no significant violation was observed. Moreover, we performed fully adjusted RCS analyses to investigate the dose‐response relationship between the CTI and the risk of new‐onset advanced CKM.

Several sensitivity analyses were performed to enhance the robustness of our study. Firstly, considering the potential influence of the cancer, participants with a history of cancer were excluded for the main analyses. Second, to minimize the influence of acute inflammatory disturbances, a sensitivity analysis was conducted after excluding participants with acute infection, severe autoimmune diseases, or recent major surgery. Third, to address potential selection bias introduced by complete case analysis, a sensitivity analysis was performed using multiple imputation (MI) to handle missing covariates. Fourth, given that death may serve as a competing event for incident advanced CKM, the Fine–Gray competing risk model was performed as a sensitivity analysis. Additionally, subgroup analyses were performed to assess whether the impact of CTI on the risk of advanced CKM varied among different groups. Participants were stratified by age, gender, smoking status, and drinking status.

R software (version 4.4.1) was employed for all statistical analyses in this study. Main analytical packages applied in this study included glmnet v4.1‐8, Boruta v8.0.0, e1071 v1.7‐16, survival v3.7‐0, and rms v8.1‐1. All tests were two‐tailed, and *p*  < 0.05 was considered statistically significant.

## 3. Results

### 3.1. Baseline Characteristics of the Study Population

For the screening analysis, a total of 8959 participants from the NHANES database were enrolled, with a mean age of 49.4 years and 50.9% females. Significant intergroup differences were observed in demographic features, lifestyle factors, anthropometric measurements, medication use, blood pressure, glycemic and lipid parameters, renal function, and inflammatory biomarkers between participants with nonadvanced and advanced CKM (Supporting Information 3: Table S2).

In the cross‐sectional analyses, 8959 participants from 208,625 from UKB (53.5% female; mean age: 56.6 years), NHANES (50.9% female; mean age: 49.4 years), and 8550 from CHARLS (54.3% female; mean age: 59.2 years) were included. Detailed characteristics are presented in Supporting Information 3: Tables S2–S4.

In the longitudinal analyses, 186,753 participants from UKB (57.5% female; mean age: 59.2 years) and 3673 from CHARLS (60.2% female; mean age: 61.2 years) were analyzed. Participants were categorized into three groups based on tertiles of CTI, and baseline characteristics according to CTI groups are summarized in Supporting Information 3: Tables S5 and S6. To evaluate potential selection bias, baseline characteristics between included and excluded participants were compared, showing no significant differences in key variables (Supporting Information 3: Table S7).

### 3.2. CTI Identified as the Optimal Inflammation Biomarker

**Figure 1:**
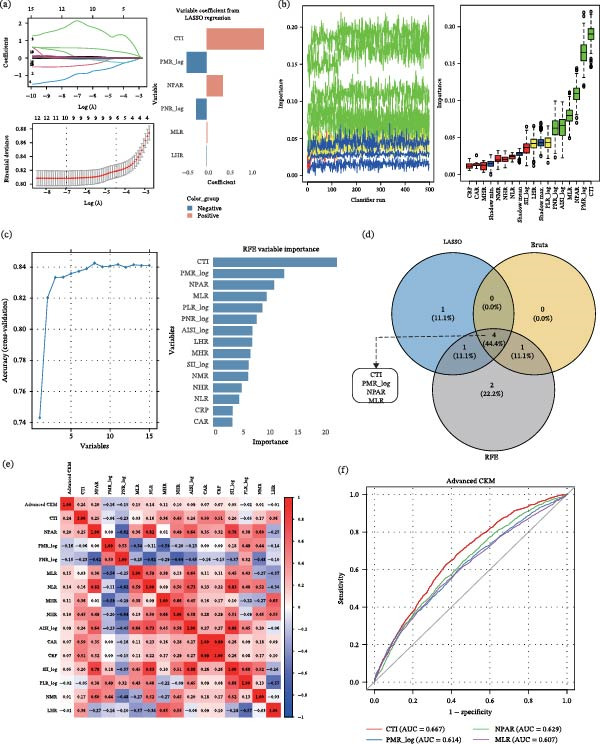
Screening for the optimal inflammation biomarker associated with the risk of advanced CKM. (a) LASSO algorithm for feature selection. (b) Boruta algorithm for feature selection. (c) SVM‐RFE algorithm for feature selection. (d) Venn plot based on outcomes from three machine learning methods. (e) Correlation analyses between 15 blood‐derived inflammation biomarkers and advanced CKM risk. CTI showed the most relevance to advanced CKM. (f) Receiver operation curves for the association between ML‐identified markers and risk of advanced CKM. Abbreviations: CKM, cardiovascular–kidney–metabolic syndrome; CTI, C‐reactive protein–triglyceride glucose index; LASSO, Least Absolute Shrinkage and Selection Operator; MLR, monocyte‐to‐lymphocyte ratio; NPAR, neutrophil percentage‐to‐albumin ratio; PMR‐log, logarithmically transformed platelet‐to‐monocyte ratio; SVM‐RFE, support vector machine–recursive feature elimination.

The distributions of 15 blood‐derived inflammatory biomarkers between participants with advanced and nonadvanced CKM are presented in Supporting Information 2: Figure S5. Three machine learning algorithms—LASSO (Figure 1a), Boruta (Figure 1b), and SVM‐RFE (Figure 1c)—were applied to identify candidate biomarkers. CTI, NPAR, PMR‐log, and MLR were consistently identified as important features across all three methods and were therefore selected as the four potential markers (Figure 1d). Among them, CTI showed the strongest correlation with advanced CKM and yielded the highest area under the ROC curve (AUC) (Figure 1e,f). Based on these findings, CTI was selected as the optimal inflammatory biomarker for subsequent analyses.

### 3.3. CTI Reflects CKM Severity Across Multiple National Cohorts

**Figure 2:**
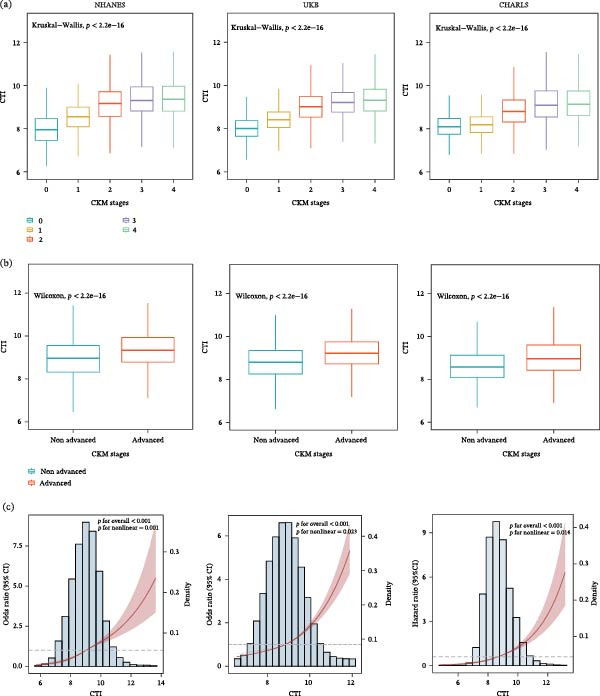
CTI levels among different CKM stages in NHANES, UKB, and CHARLS. (a) CTI levels among CKM from stages 0–4 in three cohorts. (b) CTI levels between participants with nonadvanced CKM and advanced CKM in three cohorts. (c) Restricted cubic spline analyses between CTI and risk of advanced CKM based on a cross‐sectional study in three cohorts. Abbreviations: CHARLS, China Health and Retirement Longitudinal Study; CKM, cardiovascular–kidney–metabolic syndrome; CTI, C‐reactive protein–triglyceride glucose index; NHANES, National Health and Nutrition Examination Survey; UKB, UK Biobank.

CTI values increased progressively with advancing CKM stages (0–4) in UKB (United Kingdom), NHANES (United States), and CHARLS (China) (Figure 2a). Participants with advanced CKM had significantly higher CTI levels than those with nonadvanced CKM in all three cohorts (Figure 2b). After full adjustment for potential confounders in Model 3 (Table 1), continuous CTI remained positively associated with advanced CKM (NHANES: odds ratio [OR] = 1.35, 95% confidence interval (CI): 1.20–1.48; UKB: OR = 1.55, 95% CI: 1.46–1.64; CHARLS: OR = 1.42, 95% CI: 1.28–1.60). Similarly, higher CTI tertiles were associated with an increase in advanced CKM risk compared with the lowest tertile across all three cohorts: NHANES (T2: OR = 1.18, 95% CI: 1.01–1.40; T3: OR = 1.65, 95% CI: 1.39–1.91), UKB (T2: OR = 1.28, 95% CI: 1.18–1.39; T3: OR = 2.02, 95% CI: 1.86–2.21), and CHARLS (T2: OR = 1.38, 95% CI: 1.22–1.63; T3: OR = 2.28, 95% CI: 2.01–2.63). RCS analyses further demonstrated a consistent, monotonically increasing nonlinear relationship between CTI and advanced CKM across these national cohorts (Figure 2c).

**Table 1:** Association of CTI with risk of advanced CKM based on cross‐sectional data from NHANES, UKB, and CHARLS.

	Model 1		Model 2		Model 3	
	OR (95% CI)	*p*‐Value	OR (95% CI)	*p*‐Value	OR (95% CI)	*p*‐Value
*NHANES*						
CTI continuous	1.66 (1.51–1.76)	<0.001	1.47 (1.34–1.64)	<0.001	1.35 (1.20–1.48)	<0.001
T1	Ref	—	Ref	—	Ref	—
T2	1.91 (1.59–2.19)	<0.001	1.25 (1.07–1.49)	0.013	1.18 (1.01–1.40)	0.042
T3	2.83 (2.37–3.22)	<0.001	1.87 (1.53–2.16)	<0.001	1.65 (1.39–1.91)	<0.001
* p* for trend	—	<0.001	—	<0.001	—	<0.001
*UKB*						
CTI continuous	1.89 (1.77–1.99)	<0.001	1.71 (1.65–1.79)	<0.001	1.55 (1.46–1.64)	<0.001
T1	Ref	—	Ref	—	Ref	—
T2	1.91 (1.76–2.11)	<0.001	1.41 (1.28–1.51)	<0.001	1.28 (1.18–1.39)	<0.001
T3	3.21 (2.96–3.48)	<0.001	2.37 (2.19–2.58)	<0.001	2.02 (1.86–2.21)	<0.001
* p* for trend	—	<0.001	—	<0.001	—	<0.001
*CHARLS*						
CTI continuous	1.94 (1.71–2.26)	<0.001	1.80 (1.64–2.04)	<0.001	1.42 (1.28–1.60)	<0.001
T1	Ref	—	Ref	—	Ref	—
T2	1.66 (1.43–1.99)	<0.001	1.59 (1.35–1.80)	<0.001	1.38 (1.22–1.63)	<0.001
T3	3.15 (2.71–3.56)	<0.001	2.89 (2.49–3.24)	<0.001	2.28 (2.01–2.63)	<0.001
* p* for trend	—	<0.001	—	<0.001	—	<0.001

*Note:* Model 1: Crude unadjusted model. Model 2: Adjusted for age and gender. Model 3: Further adjusted for smoking status, drinking status, BMI, SBP, DBP, LDL‐C, eGFR, antidiabetic drugs, lipid‐lowering drugs, and baseline CKM stage based on Model 2.

Abbreviations: BMI, body mass index; CHARLS, China Health and Retirement Longitudinal Study; CI, confidence interval; CKM, cardiovascular–kidney–metabolic syndrome; CTI, C‐reactive protein to triglyceride glucose index; DBP, diastolic blood pressure; eGFR, estimated glomerular filtration rate; LDL‐C, low‐density lipoprotein cholesterol; NHANES, National Health and Nutrition Examination Survey; OR, odds ratio; SBP, systolic blood pressure; UKB, UK Biobank.

### 3.4. CTI Predicts the Progression of CKM Across Multiple National Cohorts

**Figure 3:**
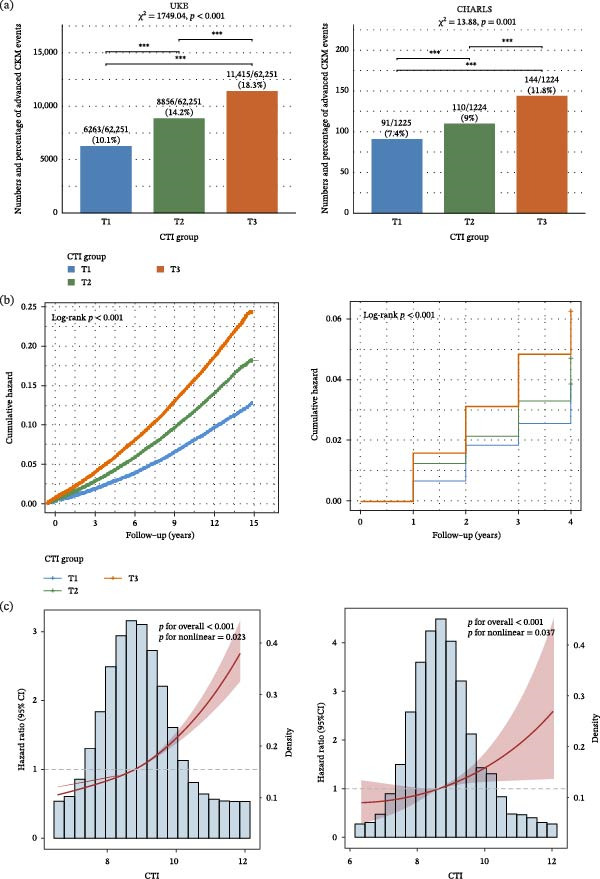
Association between CTI and new onset of advanced CKM risk based on a longitudinal study in two cohorts. (a) Numbers and percentage of new‐onset advanced CKM events among different CTI tertiles during the follow‐up in UKB and CHARLS. (b) Kaplan–Meier cumulative risk curve in UKB and CHARLS. (c) Restricted cubic spline analyses between CTI and risk of new‐onset advanced CKM based on a longitudinal study in UKB and CHARLS. Abbreviations: CHARLS, China Health and Retirement Longitudinal Study; CKM, cardiovascular–kidney–metabolic syndrome; CTI, C‐reactive protein–triglyceride glucose index; UKB, UK Biobank.

During a median follow‐up of 13.1 years in UKB and 4.0 years in CHARLS, 26,534 and 345 incident advanced CKM events were recorded, respectively. The incidents of advanced CKM increased progressively across CTI tertiles in both cohorts (UKB: T1 6263 [10.1%], T2 8856 [14.2%], and T3 11,415 [18.3%]; CHARLS: T1 91 [7.4%], T2 110 [9.0%], and T3 144 [11.8%]; Figure 3a). Kaplan–Meier analyses demonstrated a stepwise increase in cumulative hazard from T1 to T3 of CTI, with significant differences observed in both cohorts (log‐rank *p*  < 0.001; Figure 3b).

After full adjustment for potential confounders in Model 3 (Table 2), continuous CTI was positively associated with the risk of new‐onset advanced CKM (UKB: HR = 1.22, 95% CI: 1.17–1.30; CHARLS: HR = 1.21, 95% CI: 1.01–1.46). Similarly, higher CTI tertiles were associated with progressively increased risk compared with T1 (UKB: T2 HR = 1.15, 95% CI: 1.09–1.25; T3 HR = 1.43, 95% CI: 1.34–1.55; CHARLS: T2 HR = 1.26, 95% CI: 1.05–1.53; T3 HR = 1.45, 95% CI: 1.20–1.76). RCS analyses based on Cox models further confirmed a monotonically increasing, nonlinear association between CTI and incident advanced CKM in both cohorts (Figure 3c).

**Table 2:** Association of CTI with risk of new‐onset advanced CKM based on longitudinal data in UKB and CHARLS.

	Events (*n*)	Model 1		Model 2		Model 3	
		HR (95% CI)	*p*‐Value	HR (95% CI)	*p*‐Value	HR (95% CI)	*p*‐Value
*UKB*							
CTI continuous	26,534/186,753	1.45 (1.33–1.60)	<0.001	1.37 (1.31–1.45)	<0.001	1.22 (1.17–1.30)	<0.001
T1	6263/62,251	Ref	—	Ref	—	Ref	—
T2	8856/62,251	1.44 (1.32–1.51)	<0.001	1.21 (1.15–1.26)	<0.001	1.15 (1.09–1.25)	<0.001
T3	11,415/62,251	1.95 (1.82–2.05)	<0.001	1.58 (1.50–1.69)	<0.001	1.43 (1.34–1.55)	<0.001
* p* for trend	—	—	<0.001	—	<0.001	—	<0.001
*CHARLS*							
CTI continuous	345/3673	1.46 (1.15–1.74)	<0.001	1.29 (1.09–1.57)	<0.001	1.21(1.01–1.46)	0.029
T1	91/1225	Ref	—	Ref	—	Ref	—
T2	110/1224	1.57 (1.30–1.80)	<0.001	1.41 (1.11–1.66)	0.006	1.26 (1.05–1.53)	0.011
T3	144/1224	1.98 (1.62–2.39)	<0.001	1.67 (1.30–1.99)	<0.001	1.45 (1.20–1.76)	<0.001
* p* for trend	—	—	<0.001	—	<0.001	—	<0.001

*Note:* Model 1: crude unadjusted model. Model 2: Adjusted for age and gender. Model 3: Further adjusted for smoking status, drinking status, BMI, SBP, DBP, LDL‐C, eGFR, antidiabetic drugs, lipid‐lowering drugs, and baseline CKM stage based on Model 2.

Abbreviations: BMI, body mass index; CHARLS, China Health and Retirement Longitudinal Study; CI, confidence interval; CKM, cardiovascular–kidney–metabolic syndrome; CTI, C‐reactive protein to triglyceride glucose index; DBP, diastolic blood pressure; eGFR, estimated glomerular filtration rate; HR, hazard ratio; LDL‐C, low‐density lipoprotein cholesterol; NHANES, National Health and Nutrition Examination Survey; SBP, systolic blood pressure; UKB, UK Biobank.

### 3.5. Sensitivity Analyses

Subgroup analyses were carried out in both cross‐sectional and longitudinal studies (Supporting Information 2: Figures S6 and S7). Stratified by age, gender, smoking status, and drinking status, the outcomes were consistent across all subgroups, and no significant interaction was observed in different cohorts. Additionally, we conducted several sensitivity analyses to estimate the robustness of our findings. Firstly, the results remained consistent after excluding participants with a history of cancer (Supporting Information 3: Tables S8 and S9). Secondly, after excluding participants with acute infection, severe autoimmune diseases, or recent major surgery, the findings did not materially change (Supporting Information 3: Tables S10 and S11). Thirdly, the sensitivity analysis using MI to handle missing covariates yielded similar results, indicating that the influence of selection bias was minimal (Supporting Information 3: Tables S12 and S13). Fourthly, the results from the Fine–Gray competing risk model, accounting for death as a competing event, were consistent with the primary analyses (Supporting Information 3: Table S14).

## 4. Discussion

Using three nationally representative cohorts, machine learning algorithms, and cross‐cohort comparisons, we identified CTI as the optimal inflammatory biomarker for assessing CKM severity and predicting disease progression. The robustness of this finding was further confirmed through multiple sensitivity and subgroup analyses. To the best of our knowledge, this is the first study to systematically evaluate and compare a wide range of inflammatory markers to identify the most informative biomarker for CKM. Importantly, this study highlights the predictive value of CTI for CKM, providing a potential tool for clinical risk stratification and guiding management strategies.

CTI is an emerging composite index combining CRP and the triglyceride‐glucose (TyG) index. The TyG index, a validated surrogate marker for insulin resistance (IR) [23], is strongly linked to multiple cardiometabolic and renal diseases. Lopez‐Jaramillo et al. [24] reported its significant association with cardiovascular mortality, myocardial infarction, stroke, and type 2 diabetes. Fritz et al. [25] also found that the TyG index correlated with end‐stage kidney disease (ESKD) risk and mediated nearly half of the BMI‐ESKD association. As a classic biomarker of systemic inflammation, CRP is well documented to affect disease onset, progression, and prognosis [26].

By integrating indicators of IR and systemic inflammation, CTI enables comprehensive evaluation for cardiometabolic‐renal diseases. Supporting our findings, Ou et al. [27] confirmed that higher CTI predicts elevated CVD and mortality risks in middle‐aged and elderly Chinese based on the CHARLS cohort. Ma et al. [28] further demonstrated that cumulative and sustained high CTI independently increases CVD risk, providing partial evidence for our results.

Notably, effect sizes fluctuated moderately across the three cohorts, with OR estimates ranging from 1.41 to 1.63. Such a minor heterogeneity is reasonable, mainly attributed to varied population demographics and lifestyle disparities across different datasets. Most existing relevant studies have drawn conclusions consistent with our results, and few publications have presented contradictory or nonsignificant associations regarding CTI and CKM progression [26–28].

In comparison with other commonly used markers including NPAR and MLR that only reflect a single physiological abnormality, CTI integrates both inflammatory response and IR status. This dual‐dimensional construction enables CTI to capture complex interactive pathological damage, thus exhibiting superior predictive ability for CKM progression.

CKM staging reflects the continuum of cumulative organ injury. Nonadvanced CKM (stages 0–2) represents mild‐to‐moderate localized organ injury, while advanced CKM (stages 3–4) indicates multisystem impairment and markedly elevated systemic risk [5]. The mechanisms underlying CKM progression remain unclear, with IR and inflammation identified as key drivers of this transition [27, 29], which can be partially reflected by CTI levels.

As the metabolic component of CTI, TyG reflects systemic IR and mediates heart–kidney–metabolic crosstalk. IR directly contributes to metabolic dysfunction through impaired glucose uptake and lipid accumulation while simultaneously activating the renin–angiotensin–aldosterone system (RAAS) and promoting sodium retention. These metabolic perturbations collectively damage blood vessels, increase glomerular pressure, and impair cardiac energy metabolism, thereby driving multisystem metabolic and organ injury in CKM progression [30, 31].

As the inflammatory component of CTI, CRP‐related systemic inflammation concurrently promotes multiple CKM pathological lesions. Elevated inflammatory cytokines, including CRP and interleukins, induce endothelial dysfunction and accelerate atherosclerosis, contributing to myocardial ischemia and cardiac fibrosis [32]. Concurrently, inflammation activates innate immune responses, increases the release of proinflammatory cytokines such as IL‐1 and IL‐18, and promotes oxidative stress and endothelial dysfunction, ultimately triggering renal tubular cell death, glomerular damage, and renal fibrosis to accelerate CKD progression [33].

Notably, inflammation and IR are closely interconnected and mutually exacerbate each other, forming a self‐reinforcing vicious cycle that dominates CKM deterioration [34, 35]. Chronic inflammation impairs insulin signaling pathways and aggravates IR, whereas metabolic dysfunction further amplifies systemic inflammatory responses [36, 37]. Unlike individual indicators (CRP, TyG, or FPG) that only reflect a single pathological dimension, CTI integrates interactive metabolic and inflammatory pathways, comprehensively capturing the multidimensional cardiorenal‐metabolic pathophysiological characteristics of CKM. This inherent superiority enables CTI to better evaluate the CKM severity and predict progressive organ damage.

Our study possesses several notable strengths. First, we analyzed three nationally representative databases, yielding consistent findings that underscore the robustness of our results. Second, we comprehensively examined the relationship between CTI and advanced CKM risk employing both cross‐sectional and longitudinal analyses, enhancing the reliability of our conclusions. Third, we conducted an extensive series of sensitivity and subgroup analyses, further reinforcing the robustness of the primary findings.

Nevertheless, this study is subject to certain limitations. First, self‐reported disease data may cause nondifferential misclassification, which could underestimate the real association effects. Our results are therefore conservative, and the actual predictive potency of CTI may be higher. Second, the CHARLS cohort had a relatively small sample of 3673 participants. Even so, significant associations were observed, and the effect trend matched the UKB data, verifying result reliability. Third, several unadjusted factors including dietary pattern, physical activity level, genetic background, and anti‐inflammatory medications may alter inflammation and IR status, possibly confounding the correlation between CTI and CKM risk. Fourth, ML‐based feature selection was performed in the NHANES cross‐sectional dataset rather than in a longitudinal dataset. Although CTI was validated in longitudinal cohorts, biomarkers associated with prevalent diseases may not be optimal for predicting prospective progression. Future longitudinal feature selection studies are warranted to confirm the predictive value of CTI. Fifth, the primary Cox model does not account for death as a competing risk. Although the Fine–Gray model confirmed robust results, this represents a potential methodological limitation. Sixth, although participants were excluded from the longitudinal analyses according to prespecified criteria, potential selection bias or bias related to missing data cannot be entirely ruled out. Seventh, the CTI index, comprising CRP, TG, and FPG, partially overlaps with CKM components, potentially inflating the observed associations and necessitating cautious interpretation. Finally, only baseline CTI data were analyzed in this study. Subsequent research should explore the dynamic variation and cumulative exposure of CTI to improve its predictive efficacy for CKM.

From the perspective of clinical application, CTI features easy calculation and low testing cost, relying only on routine clinical indicators for assessment. It can be effectively used for large‐scale population screening and identify individuals at high risk of CKM progression, so as to facilitate targeted monitoring and early intervention. Given its simplicity and accessibility, CTI is particularly suitable for risk evaluation and hierarchical disease management in primary healthcare settings and resource‐limited regions, where complex professional risk scoring systems are difficult to popularize and implement.

## 5. Conclusion

In this study of three nationally representative cohorts, CTI emerged as the optimal inflammatory biomarker of advanced CKM among multiple candidates. Elevated CTI levels were consistently associated with an increased risk of advanced CKM in both cross‐sectional and longitudinal analyses. These findings highlight its potential utility for assessing disease severity and predicting CKM progression, offering a basis to refine CKM management.

**Nomenclature**
CKM: Cardiovascular–kidney–metabolic syndrome
CTI: C‐reactive protein–triglyceride glucose index
UKB: UK Biobank
NHANES: National Health and Nutrition Examination Survey
CHARLS: China Health and Retirement Longitudinal Study.

## Author Contributions

Yupeng Zeng designed the study and wrote the original draft. Huhao Feng and Haiying Cheng performed the main analyses. Hui He performed data curation and initiated the tables and figures. Weiqing Wu, Lixin Cheng, and Qingshan Geng supervised the study and revised the manuscript.

## Funding

This research was supported by the National Natural Science Foundation of China (Grants 62472207 and 32370711), the Major Science and Technology Special Project of Henan Province (Grant 241100310300), and the Shenzhen Medical Research Fund (Grant A2303033).

## Ethics Statement

The NHANES, UKB, and CHARLS were approved by the National Center for Health Statistics Research Ethics Review Board, North West Multi‐center Research Ethics Committee, and Ethics Review Committees of Peking University, respectively. All studies fulfilled all principles of the Declaration of Helsinki. Additionally, written informed consent was obtained from each participant in these three cohorts. As such, no further ethical review or approval was required for this research.

## Conflicts of Interest

The authors declare no conflicts of interest.

## Supporting Information

Additional supporting information can be found online in the Supporting Information section.

## Supporting information


**Supporting Information 1** Supporting methods.

**Supporting Information 2** Supporting figures.

**Supporting Information 3** Supporting tables.

## Data Availability

Data from NHANES are publicly available at https://www.cdc.gov/nchs/NHANES/. Data from UKB are publicly available to bona fide researchers upon application at https://www.ukbiobank.ac.uk. All data used in this study were accessed from the UK Biobank under Application Number 662558. Data from CHARLS are publicly available at https://charls.charlsdata.com/pages/data/111/zh-cn.html.
