# Supplementary material for: Integrative Inflammation–Metabolism Indicator for Cardiovascular–Kidney–Metabolic Syndrome: Evaluating the C‐Reactive Protein–Triglyceride Glucose Index for Risk Stratification and Progression Across Three National Cohorts
Source: Mediators Inflamm. 2026 Jul 28;2026:8912366. doi: 10.1155/mi/8912366 (PMC13410279; doi:10.1155/mi/8912366)
Supplement: Supplementary file 1 — Supporting Information 1 Supporting methods. [file MI-2026-8912366-s002.pdf]

# Supplementary Methods

**Details of the three cohorts included in this study**

**National Health and Nutrition Examination Survey**

The National Health and Nutrition Examination Survey (NHANES) is a main project of National Center for Health Statistics (NCHS) designed to investigate the nutritional and health status among American noninstitutionalized population. NHANES combines interviews, physical examinations, and laboratory tests to collect data on a wide range of health indicators, including dietary habits, chronic conditions, infectious diseases, and environmental exposures. The survey employs a stratified, complex, multistage probability sampling design to ensure nationally representative results. The whole study was authorized by Research Ethic Review Board of NCHS, and all the respondents signed the written consent forms.

**UK biobank**

The UK Biobank (UKB) is a population-based cohort that involves over 500,000 individuals aged 40–69 years old recruited from 22 centers across the United Kingdom between 2006 and 2010. During the initial assessment phase, participants completed a digital, self-administered touchscreen survey and took part in computer-assisted personal interviews. Biological samples were also collected as a component of the thorough evaluation process. Self-reported information covered demographic background, smoking habits, alcohol consumption, past medical conditions, and current medication use; all health-related histories were cross-verified through interviewer-led validation. Subject to consent, these core data were subsequently linked to electronic health records, including hospital admissions, mortality registries, and primary care documentation. The ethical approval was obtained from the National Information Governance Board for Health and Social Care and the North West Multi-Centre Research Ethics Committee (11/NW/0382). All participants signed written informed consent form.

**China Health and Retirement Longitudinal Study**

The China Health and Retirement Longitudinal Study (CHARLS) is a nationally representative longitudinal survey tracking the health, socioeconomic status, and well-being of China's middle-aged and older population. Initiated in 2011/2012 with biennial follow-ups, CHARLS employs a multistage probability sampling strategy covering approximately 150 counties/districts to ensure national representativeness. The survey covers a wide range of topics, including demographic background,

family structure, health status, healthcare utilization, employment, income, and assets. Its biennial follow-up design enables researchers to analyze dynamic changes in individuals' lives over time. The ethical approval was obtained from Ethics Review Committees of Peking University. Informed consent was obtained from each participant.

**Definition of CKM syndrome**

CKM syndrome characterizes the complex interplay between CVD, CKD and metabolic dysregulation. CKM stages (0-4) were classified according to the American Heart Association (AHA) Presidential Advisory framework, which delineates a spectrum of escalating cardiometabolic and renal risk. Stage 0: Denotes absence of CKM risk factors. Participants met criteria for normal adiposity (BMI <25 kg/m² [<23 kg/m² for Asian individuals], waist circumference <102/88 cm for men/women [<90/80 cm for Asian men/women]), lacked metabolic risk factors (hypertension, hypertriglyceridemia, metabolic syndrome, diabetes, prediabetes), and had no evidence of CKD. Stage 1: Defined by the presence of excess or dysfunctional adiposity, indicated by elevated BMI (⩾25 kg/m² [⩾23 kg/m² for Asian individuals]) or waist circumference (⩾102/88 cm for men/women [⩾90/80 cm for Asian men/women]), or prediabetes. No metabolic risk factors or CKD were present. Stage 2: Characterized by established metabolic risk factors (hypertriglyceridemia, hypertension, metabolic syndrome, or diabetes) and/or CKD categorized as moderate-to-high risk based on KDIGO guidelines. CKD risk was stratified using estimated glomerular filtration rate (eGFR, calculated via the CKD-EPI creatinine equation). Stage 3: Included individuals without diagnosed clinical CVD but possessing either a high predicted 10-year CVD risk (assessed using the AHA PREVENT equations) or very-high-risk CKD. Stage 4: Encompassed individuals with existing clinical CVD (coronary heart disease, heart failure, stroke, peripheral artery disease, or atrial fibrillation).

**Details of the covariates**

Due to different designs and data availability across the three cohorts, covariates differ somewhat. The education levels in NHANES and CHARLS were divided into three groups: less than high school, high school, and above high school; while in UKB, the education levels were categorized into: unknow, college, and other levels. Smoking status and drinking status were categorized into never, or former/current in all three cohorts. Use of antidiabetic and lipid-lowering drugs was categorized as yes or no across three cohorts.

**Details of machine learning algorithms included in this study**

The Boruta algorithm is a robust feature selection method based on random forests methodology. It identifies significant features by comparing their importance against artificially created shadow features, thereby reducing model error and identifying the optimal feature subset. LASSO shrinks some coefficients to exactly zero by applying L1 regularization, effectively excluding irrelevant features, enabling the selection of the most predictive features. SVM-RFE, a feature elimination method based on support vector machines, was used to iteratively remove the least important features, thereby refining the set of key inflammation biomarkers that contribute to progression of the stages of CKM.

**Definition of diseases and medical conditions included in this study**

**Cardiovascular disease**

Cardiovascular disease (CVD) in this study included coronary heart disease, heart failure, stroke, atrial fibrillation. CVD was defined by International Classification of Diseases and Related Health Problems, 10th Revision (ICD-10) codes (I20-I25, I48, I50, I60-I64, I69), questionnaire of self-reported medical conditions of CVD.

**Type2 diabetes mellitus**

Type2 diabetes mellitus (T2DM) was defined as fulfilling anyone of the following criteria: (1) ICD-10 code (E11), (2) fasting plasma glucose levels ⩾7.0 or random glucose levels ⩾11.1 mmol/L from laboratory test, (3) the use of antidiabetic medications, (4) questionnaire of self-reported medical conditions of T2DM.

**Prediabetes**

Prediabetes is defined as a condition where blood glucose levels are higher than normal but not yet high enough to be diagnosed as diabetes. It serves as an early warning stage for diabetes. The specific diagnostic criteria are as follows: (1) fasting plasma glucose levels between 6.1 mmol/L (110 mg/dL) and 6.9 mmol/L (125 mg/dL); (2) during an oral glucose tolerance test (OGTT), 2-hour plasma glucose levels between 7.8 mmol/L (140 mg/dL) and 11.0 mmol/L (199 mg/dL), (3) Glycated Hemoglobin (HbA1c): HbA1c levels between 5.7% and 6.4%.

**Hypertension**

Hypertension was defined as meeting anyone of the following criteria: (1) ICD-10 code (I10), (2) systolic blood pressure ≥140 mmHg or diastolic pressure ≥90 mmHg, (3) the use of antihypertensive drugs, (4) questionnaire of self-reported medical conditions of hypertension.

**Chronic kidney disease**

Chronic kidney disease (CKD) was defined as meeting anyone of the following criteria: (1) ICD-10 code (N18), (2) estimated glomerular filtration rate (eGFR) < 60ml/min, (3) questionnaire of self-reported medical conditions of hypertension of CKD.

**Metabolic syndrome**

Metabolic syndrome (MetS) was defined as meeting three or more of the following criteria: (1) waist circumference ≥102/88 cm in men/women (≥90/80 cm for Asian men/women); (2) high circulating triglycerides (TG) ≥150 mg/dL; (3) high high-density lipoprotein cholesterol (HDL-C) < 40 mg/ dL for men and < 50 mg/dL for women; (4) high fasting blood glucose ≥ 110 mg/dL; (5) diagnosis of arterial hypertension (≥ 130/ ≥ 80 mmHg).

**Medication information**

Medication use was determined based on self-reported questionnaire information. Participants who reported regular use of hypoglycemic or lipid-regulating medications were classified as corresponding medication users.

**Statistical analyses**

All the characteristics were presented as mean (SD) for continuous variables and as frequencies and proportions for categorical variables. All continuous covariates were directly included in regression analysis. For categorical variables, never smoking, never alcohol drinking, education level below high school, non-medication use and CKM stage 0 were defined as unified reference groups in regression analyses.
