# Supplementary material for: Integrative Inflammation–Metabolism Indicator for Cardiovascular–Kidney–Metabolic Syndrome: Evaluating the C‐Reactive Protein–Triglyceride Glucose Index for Risk Stratification and Progression Across Three National Cohorts
Source: Mediators Inflamm. 2026 Jul 28;2026:8912366. doi: 10.1155/mi/8912366 (PMC13410279; doi:10.1155/mi/8912366)
Supplement: Supplementary file 2 — Supporting Information 2 Supporting figures. [file MI-2026-8912366-s003.pdf]

Supplementary Figures

Contents:

Fig. S1 Workflow of the study design.

Fig. S2 Flow chart of the study population selection in NHANES.

Fig. S3 Flow chart of the study population selection in UK biobank.

Fig. S4 Flow chart of the study population selection in CHARLS.

Fig. S5 Distribution of the Inflammation biomarkers between participants with non-advanced CKM and advanced CKM in NHANES.

Fig. S6 Subgroup analyses for association between CTI and advanced CKM risk based on cross-sectional study across three cohorts.

Fig. S7 Subgroup analyses for association between CTI and the new-onset advanced CKM risk based on longitudinal study in UKB and CHARLS.

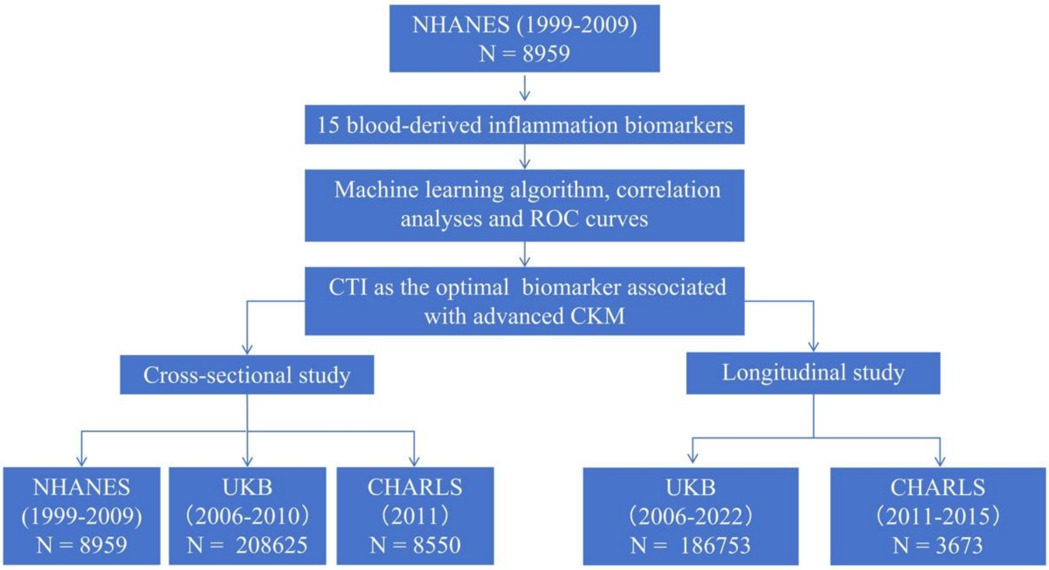

Fig. S1 Workflow of the study design.

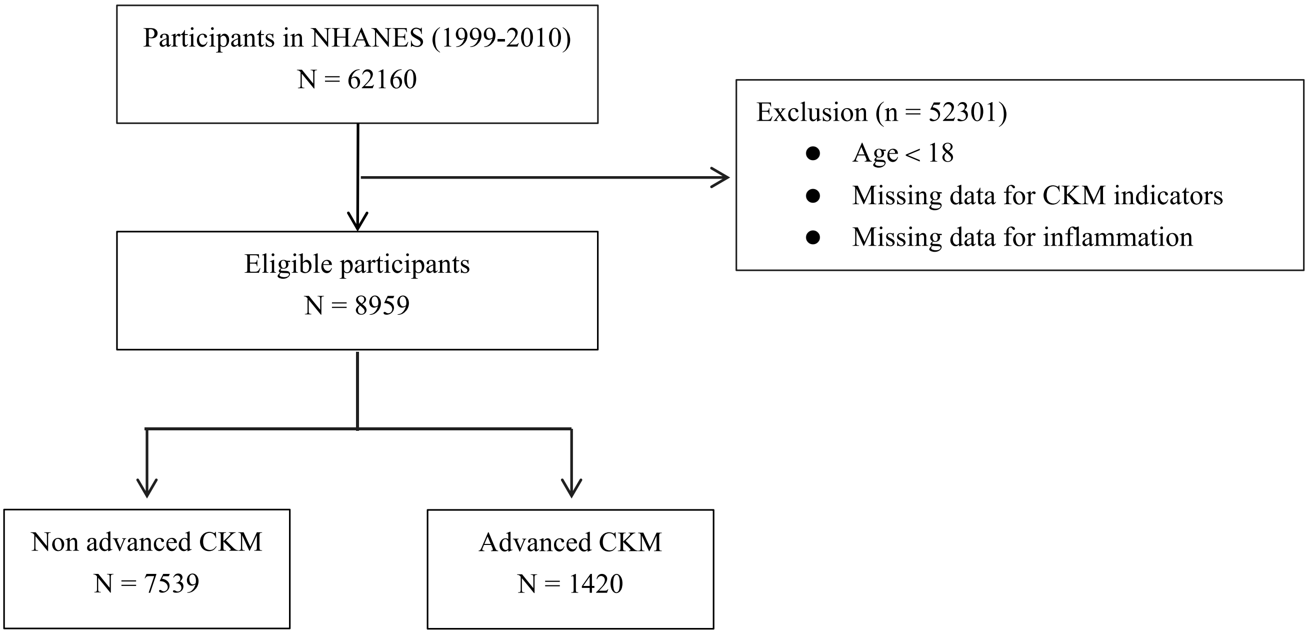

Fig. S2 Flow chart of the study population selection in NHANES

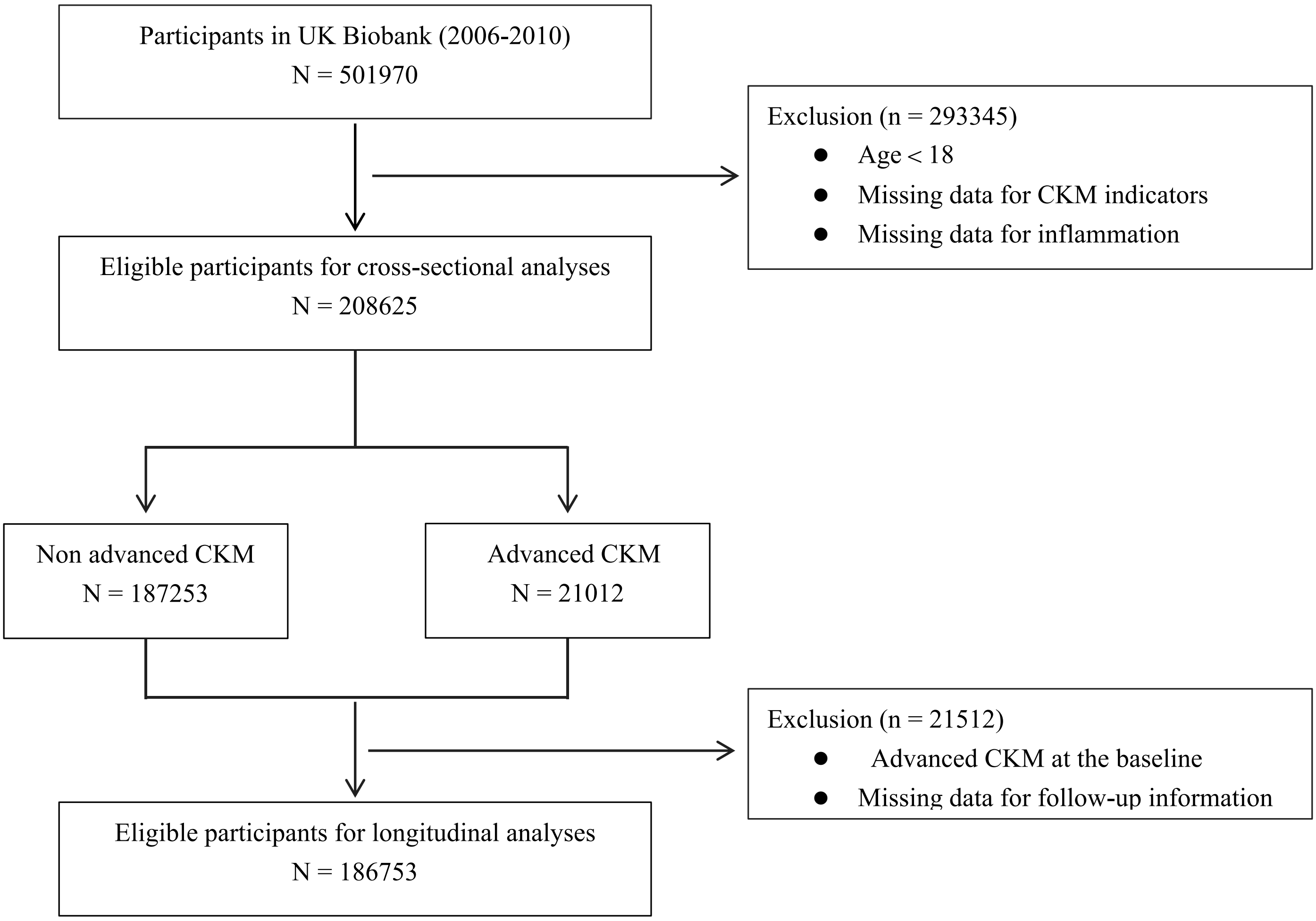

Fig. S3 Flow chart of the study population selection in UK biobank

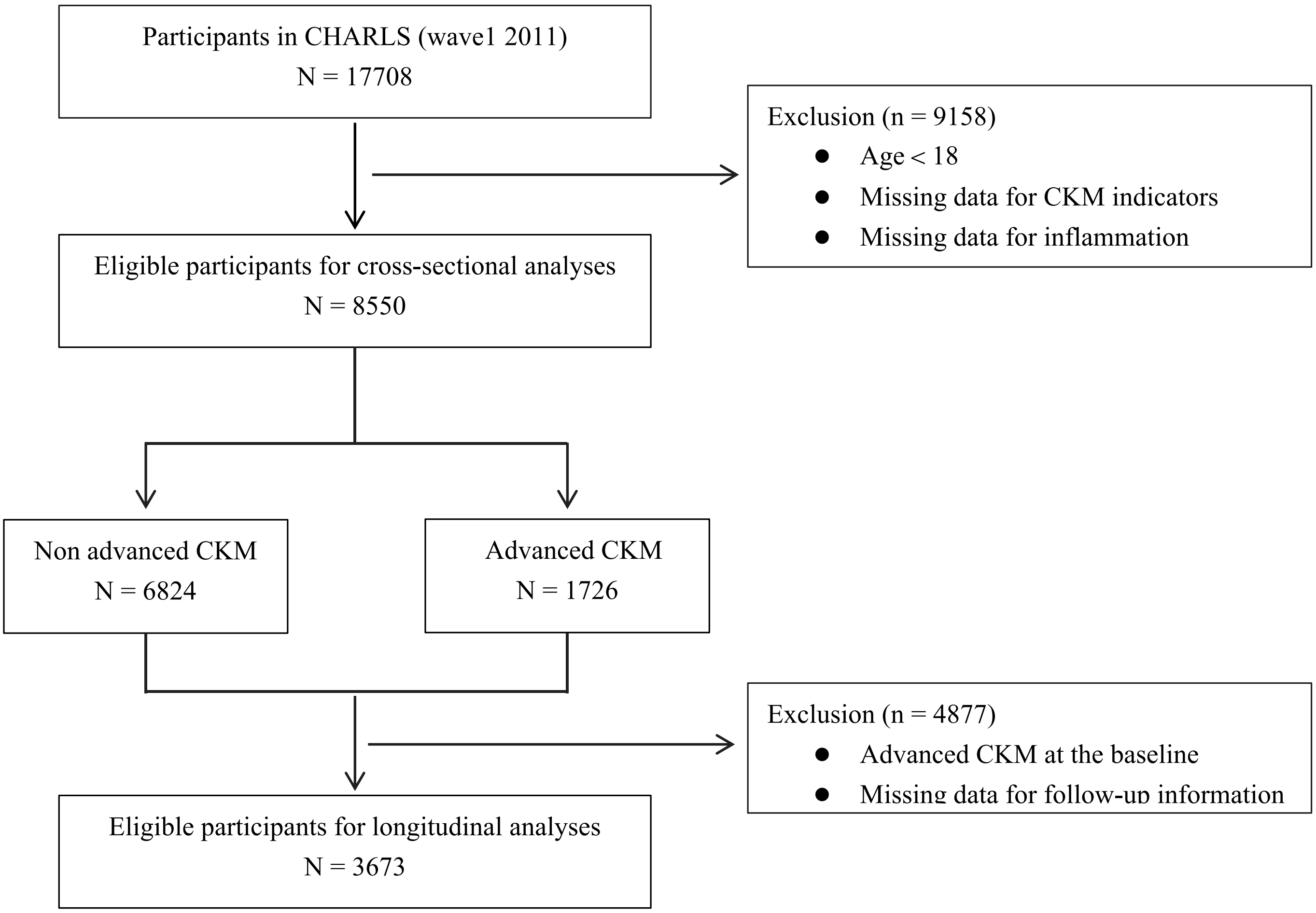

Fig. S4 Flow chart of the study population selection in CHARLS

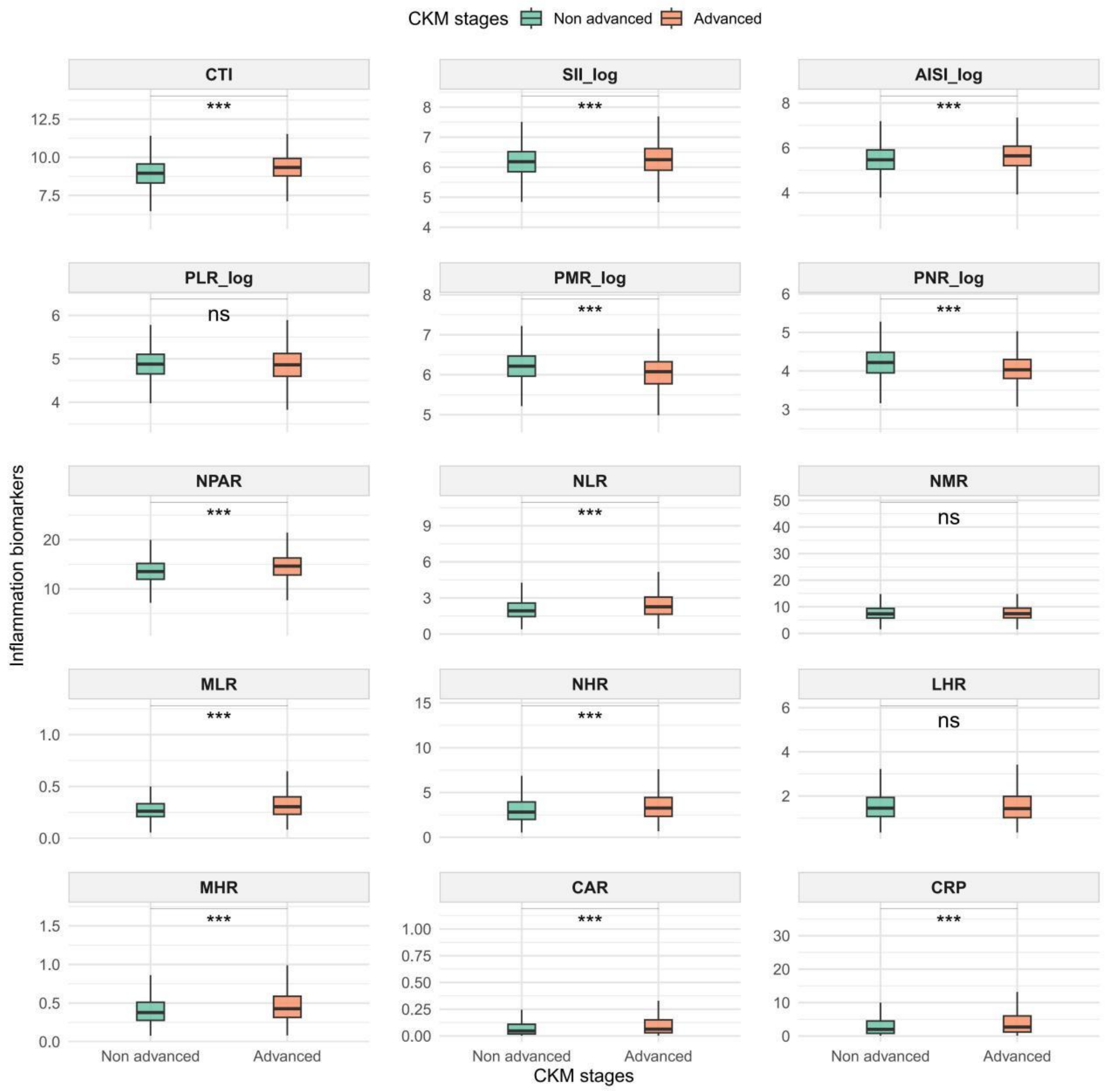

Fig. S5 Distribution of the Inflammation biomarkers between participants with non-advanced CKM and advanced CKM in NHANES. Abbreviations: CTI= C-reactive protein-triglyceride glucose index, CKM= cardiovascular-kidney–metabolic syndrome, NHANES= National Health and Nutrition Examination Survey, SII-log= logarithmically transformed systemic immune inflammation index, AISI-log= logarithmically transformed aggregate index of systemic inflammation, PLR-log= logarithmically transformed platelet to lymphocyte ratio, PMR-log= logarithmically transformed platelet to monocyte ratio, PNR-log= logarithmically transformed platelet to neutrophil ratio, NPAR= neutrophil percentage to albumin ratio, NLR= neutrophil to lymphocyte ratio, NMR= neutrophil to monocyte ratio, MLR= monocyte to lymphocyte ratio, NHR= neutrophil to high-density lipoprotein cholesterol ratio, LHR= leukocyte to high-density lipoprotein cholesterol ratio, MHR= monocyte to high-density lipoprotein cholesterol ratio, CAR= C-reactive protein to albumin ratio, CRP= C-reactive protein.

ns means no statistical significance, * means p<0.05, ** means p<0.01, *** means p<0.001

NHANES

Variable	Count	OR (95% CI)	*P* value	*P* for interaction
**Age**				0.268
<60	5954	1.52 (1.37 to 1.70)	<0.001	
>=60	3005	1.44 (1.23 to 1.67)	<0.001	
**Gender**				0.086
Female	4561	1.77 (1.62 to 1.95)	<0.001	
Male	4398	1.58 (1.45 to 1.72)	<0.001	
**Smoking status**				0.139
Never	4686	1.73 (1.57 to 1.90)	<0.001	
Former/Current	4273	1.57 (1.44 to 1.71)	<0.001	
**Drinking status**				0.498
Never	2601	1.62 (1.46 to 1.80)	<0.001	
Former/Current	6358	1.66 (1.54 to 1.80)	<0.001	

UKB

Variable	Count	OR (95% CI)	*P* value	*P* for interaction
**Age**				0.101
<60	117164	1.69 (1.61 to 1.79)	<0.001	
>=60	91101	1.63 (1.56 to 1.72)	<0.001	
**Gender**				0.226
Female	111384	1.74 (1.69 to 1.81)	<0.001	
Male	96881	1.69 (1.63 to 1.76)	<0.001	
**Smoking status**				0.381
Never	113673	1.78 (1.73 to 1.85)	<0.001	
Former/Current	94592	1.82 (1.74 to 1.90)	<0.001	
**Drinking status**				0.395
Never	16461	1.71 (1.66 to 1.78)	<0.001	
Former/Current	191804	1.75 (1.68 to 1.82)	<0.001	

CHARLS

Variable	Count	OR (95% CI)	*P* value	*P* for interaction
**Age**				0.177
<60	4702	1.81 (1.64 to 2.01)	<0.001	
>=60	3848	1.65 (1.51 to 1.81)	<0.001	
**Gender**				0.152
Female	4645	1.66 (1.51 to 1.83)	<0.001	
Male	3905	1.83 (1.67 to 2.01)	<0.001	
**Smoking status**				0.349
Never	5248	1.68 (1.54 to 1.84)	<0.001	
Former/Current	3302	1.80 (1.63 to 1.98)	<0.001	
**Drinking status**				0.644
Never	5050	1.68 (1.54 to 1.83)	<0.001	
Former/Current	3500	1.73 (1.57 to 1.90)	<0.001	

Fig. S6 Subgroup analyses for association between CTI and advanced CKM risk based on cross-sectional study across three cohorts. Abbreviations: CTI= C-reactive protein-triglyceride glucose index, CKM= cardiovascular-kidney–metabolic syndrome, NHANES= National Health and Nutrition Examination Survey, UKB=UK biobank, CHARLS= China Health and Retirement Longitudinal Study, OR= odds ratio, CI= confidence interval.

UKB

Variable	Count	HR (95% CI)	*P* value	*P* for interaction
**Age**				0.314
<60	112623	1.51 (1.43 to 1.61)	<0.001	
>=60	74130	1.47 (1.39 to 1.55)	<0.001	
**Gender**				0.128
Female	107332	1.53 (1.47 to 1.58)	<0.001	
Male	79421	1.48 (1.41 to 1.57)	<0.001	
**Smoking status**				0.357
Never	106305	1.46 (1.40 to 1.53)	<0.001	
Former/Current	80448	1.42 (1.35 to 1.49)	<0.001	
**Drinking status**				0.603
Never	14348	1.44 (1.36 to 1.53)	<0.001	
Former/Current	172405	1.46 (1.39 to 1.54)	<0.001	

CHARLS

Variable	Count	HR (95% CI)	*P* value	*P* for interaction
**Age**				0.385
<60	2322	1.36 (1.17 to 1.58)	<0.001	
>=60	1398	1.24 (1.08 to 1.42)	0.002	
**Gender**				0.261
Female	2223	1.39 (1.21 to 1.60)	<0.001	
Male	1497	1.24 (1.08 to 1.43)	0.003	
**Smoking status**				0.217
Never	2527	1.46 (1.28 to 1.65)	<0.001	
Former/Current	1193	1.33 (1.16 to 1.53)	<0.001	
**Drinking status**				0.183
Never	2279	1.41 (1.22 to 1.60)	<0.001	
Former/Current	1441	1.27 (1.11 to 1.46)	<0.001	

Fig. S7 Subgroup analyses for association between CTI and the new-onset advanced CKM risk based on longitudinal study in UKB and CHARLS. Abbreviations: CTI= C-reactive protein-triglyceride glucose index, CKM= cardiovascular-kidney–metabolic syndrome, UKB=UK biobank, CHARLS= China Health and Retirement Longitudinal Study, HR= hazard ratio, CI= confidence interval.
