# Supplementary material for: Integrative Inflammation–Metabolism Indicator for Cardiovascular–Kidney–Metabolic Syndrome: Evaluating the C‐Reactive Protein–Triglyceride Glucose Index for Risk Stratification and Progression Across Three National Cohorts
Source: Mediators Inflamm. 2026 Jul 28;2026:8912366. doi: 10.1155/mi/8912366 (PMC13410279; doi:10.1155/mi/8912366)
Supplement: Supplementary file 3 — Supporting Information 3 Supporting tables. [file MI-2026-8912366-s001.pdf]

Supplementary Tables

Contents:

Table S1 Details of definition of CKM syndrome stages.

Table S2 Baseline characteristics of participants in NHANES.

Table S3 Baseline characteristics of participants for cross-sectional analyses in UK Biobank.

Table S4 Baseline characteristics of participants for cross-sectional analyses in CHARLS.

Table S5 Baseline characteristics of participants for longitudinal analyses in UK Biobank.

Table S6 Baseline characteristics of participants for longitudinal analyses in CHARLS.

Table S7 Baseline characteristics comparison between included and excluded participants for longitudinal analysis (UKB and CHARLS).

Table S8 Association of CTI with risk of advanced CKM based on cross-sectional data after excluding participants with cancer.

Table S9 Association of CTI with risk of new-onset advanced CKM based on longitudinal data after excluding participants with cancer.

Table S10 Association of CTI with risk of advanced CKM based on cross-sectional data from NHANES, UKB, and CHARLS after excluding participants with acute infection, severe autoimmune diseases, or recent major surgery.

Table S11 Association of CTI with risk of new-onset advanced CKM based on longitudinal data in UKB and CHARLS after excluding participants with acute infection, severe autoimmune diseases, or recent major surgery.

Table S12 Association of CTI with risk of advanced CKM based on cross-sectional data from NHANES, UKB, and CHARLS using multiple imputation data.

Table S13 Association of CTI with risk of new-onset advanced CKM based on longitudinal data in UKB and CHARLS using multiple imputation data.

Table S14 Association of CTI with risk of new-onset advanced CKM based on longitudinal data in UKB and CHARLS using the competing risk model.

Table S1 Details of definition of CKM syndrome stages

CKM syndrome stages	Definition
stage 0	Normal Body mass index (BMI): BMI <25 kg/m² (<23 kg/m² for Asian individuals);<br>Normal waist circumference:waist circumference<102/88 cm for men/women (<90/80 cm for Asian men/women);<br>Normal glucose level;<br>Normal lipid level;<br>Absence of CKD and CVD.
stage 1	Individuals with overweight/obesity, abdominal obesity, or dysfunctional adipose tissue.<br>BMI ≥25 kg/m2 (or ≥23 kg/m2 if Asian ancestry),<br>Waist circumference ≥88/102 cm in women/ men (or if Asian ancestry ≥80/90 cm in women/ men),<br>Fasting blood glucose ≥100–124 mg/dL or HbA1c between 5.7% and 6.4%
stage 2	Individuals with metabolic risk factors： Hypertriglyceridemia (≥135 mg/dL);<br>Hypertension (systolic blood pressure ≥140 mm Hg or diastolic blood pressure ≥90 mm Hg and/or use of antihypertensive medications);<br>Metabolic syndrome;<br>Diabetes.<br>Individuals with CKD.
stage 3	Very high-risk CKD (stage G4 or G5 CKD or very high risk per KDIGO classification)<br>High predicted 10-year CVD risk
stage 4	Individuals with clinical cardiovascular disease (coronary heart disease, HF, stroke, peripheral artery disease, atrial fibrillation) alongside with dysfunctional adiposity, other CKM risk factors, or CKD

Abbreviations: CKM= Cardiovascular-Kidney-Metabolic Syndrome, BMI= body mass index, CKD= chronic kidney disease, CVD= cardiovascular disease, KDIGO= Kidney Disease: Improving Global Outcomes.

Table S2 Baseline characteristics of participants in NHANES

Characteristic	Total<br>N = 8959	Non advanced CKM<br>N = 7539	Advanced CKM<br>N = 1420	*P* value
Age	49.4 (18.0)	46.2 (16.8)	66.6 (14.0)	<0.001
Gender				<0.001
Female	4561 (50.9%)	3898 (51.7%)	663 (46.7%)	
Male	4398 (49.1%)	3641 (48.3%)	757 (53.3%)	
Education				<0.001
Less than high school	2720 (30.4%)	2137 (28.3%)	583 (41.1%)	
High school	2128 (23.8%)	1791 (23.8%)	337 (23.7%)	
Above high school	4111 (45.9%)	3611 (47.9%)	500 (35.2%)	
Drinking status				<0.001
Never	2601 (29.0%)	2084 (27.6%)	517 (36.4%)	
Former/Current	6358 (71.0%)	5455 (72.4%)	903 (63.6%)	
Smoking status				<0.001
Never	4686 (52.3%)	4061 (53.9%)	625 (44.0%)	
Former/Current	4273 (47.7%)	3478 (46.1%)	795 (56.0%)	
BMI (kg/m²)	28.5 (6.1)	28.3 (6.0)	29.3 (6.4)	<0.001
Waist (cm)	97.8 (15.1)	96.9 (14.9)	102.8 (15.0)	<0.001
Antidiabetic drugs				<0.001
No	7839 (87.5%)	6920 (91.8%)	919 (64.8%)	
Yes	1120 (12.5%)	619 (8.2%)	501 (35.2%)	
Lipid-lowering drugs				<0.001
No	7320 (81.7%)	6447 (85.5%)	873 (61.5%)	
Yes	1639 (18.3%)	1092 (14.5%)	547 (38.5%)	
SBP (mmHg)	122.3 (18.2)	118.5 (16.3)	141.2 (20.1)	<0.001
DBP (mmHg)	71.2 (10.3)	70.1 (9.2)	78.3 (11.5)	<0.001
FPG (mg/dL)	106.4 (34.6)	103.6 (30.9)	121.4 (46.9)	<0.001
HbA1c (%)	5.6 (1.0)	5.6 (0.9)	6.1 (1.3)	<0.001
TG (mg/dL)	142.0 (106.9)	138.8 (106.3)	158.9 (108.8)	<0.001
HDL-C (mg/dL)	52.9 (15.3)	53.2 (15.2)	51.1 (15.6)	<0.001
LDL-C (mg/dL)	117.0 (35.1)	118.3 (32.4)	110.3 (40.2)	<0.001
TC (mg/dL)	198.3 (41.6)	199.3 (40.8)	193.2 (45.4)	<0.001
eGFR (mL/min/1.73m²)	98.2 (22.3)	105.1 (18.2)	65.3 (25.1)	<0.001
White blood cell count	6.8 (2.3)	6.7 (2.0)	7.2 (3.3)	<0.001
Neutrophil count	4.0 (1.6)	4.0 (1.6)	4.3 (1.6)	<0.001
Neutrophil percentage	58.1 (9.7)	57.7 (9.6)	60.2 (9.8)	<0.001
Monocyte count	0.5 (0.2)	0.5 (0.2)	0.6 (0.2)	<0.001
Lymphocyte count	2.0 (0.6)	2.0 (0.6)	1.9 (0.7)	<0.001
C-reactive protein (mg/L)	4.1 (5.7)	3.9 (5.6)	5.0 (6.5)	<0.001

Abbreviations: NHANES = National Health and Nutrition Examination Survey, CKM= Cardiovascular-Kidney-Metabolic Syndrome, BMI= body mass index, SBP= systolic blood pressure, DBP= diastolic blood pressure, FPG= fasting plasma glucose, HbA1c= glycosylated hemoglobin, TG= total triglycerides, HDL-C= high-density lipoprotein cholesterol, LDL-C= low-density lipoprotein cholesterol, TC= total cholesterol, eGFR= estimated glomerular filtration rate.

Table S3 Baseline characteristics of participants for cross-sectional analyses in UK Biobank

Characteristic	Total<br>N = 208265	Non advanced CKM<br>N = 186753	Advanced CKM<br>N = 21512	*p* value
Age	56.6 (8.1)	55.9 (8.0)	62.9 (5.4)	<0.001
Gender				<0.001
Female	111384 (53.5%)	107332 (57.5%)	4052 (18.8%)	
Male	96881 (46.5%)	79421 (42.5%)	17460 (81.2%)	
Education				<0.001
Unknown	38320 (18.4%)	31262 (16.7%)	7058 (32.8%)	
College	65473 (31.4%)	60832 (32.6%)	4641 (21.6%)	
Other levels	104472 (50.2%)	94659 (50.7%)	9813 (45.6%)	
Drinking status				<0.001
Never	16461 (7.9%)	14348 (7.7%)	2113 (9.8%)	
Former/Current	191804 (92.1%)	172405 (92.3%)	19399 (90.2%)	
Smoking status				<0.001
Never	113673 (54.6%)	106305 (56.9%)	7368 (34.3%)	
Former/Current	94592 (45.4%)	80448 (43.1%)	14144 (65.7%)	
BMI (kg/m²)	27.4 (4.7)	27.3 (4.7)	29.0 (4.7)	<0.001
Waist (cm)	90.4 (13.4)	89.4 (13.2)	98.8 (12.6)	<0.001
Antidiabetic drugs				<0.001
No	189523 (91.0%)	178642 (95.7%)	10881 (50.6%)	
Yes	18742 (9.0%)	8111 (4.3%)	10631 (49.4%)	
Lipid-lowering drugs				<0.001
No	160234 (76.9%)	150123 (80.4%)	10111 (47.0%)	
Yes	48031 (23.1%)	36630 (19.6%)	11401 (53.0%)	
SBP (mmHg)	121.5 (15.2)	119.8 (14.5)	136.2 (16.8)	<0.001
DBP (mmHg)	70.2 (9.5)	69.5 (9.2)	76.8 (10.3)	<0.001
FPG (mg/dL)	91.7 (17.1)	91.0 (16.1)	97.4 (23.5)	<0.001
HbA1c (%)	5.5 (0.6)	5.4 (0.6)	5.8 (0.9)	<0.001
TG (mg/dL)	153.9 (85.1)	150.5 (83.4)	183.3 (93.7)	<0.001
HDL-C (mg/dL)	55.9 (14.4)	56.8 (14.3)	47.4 (12.0)	<0.001
LDL-C (mg/dL)	92.6 (32.1)	94.2 (31.5)	78.9 (35.2)	<0.001
TC (mg/dL)	179.3 (36.6)	181.1 (35.7)	163.0 (40.3)	<0.001
eGFR (mL/min/1.73m²)	96.5 (18.2)	102.3 (16.5)	68.2 (22.1)	<0.001
White blood cell count	6.9 (2.0)	6.8 (2.0)	7.5 (2.2)	<0.001
Neutrophil count	4.2 (1.3)	4.2 (1.3)	4.7 (1.4)	<0.001
Neutrophil percentage	60.9 (8.4)	60.8 (8.4)	62.1 (8.8)	<0.001
Monocyte count	0.5 (0.2)	0.5 (0.2)	0.5 (0.2)	<0.001
Lymphocyte count	1.9 (0.6)	1.9 (0.6)	2.0 (0.6)	<0.001
C-reactive protein (mg/L)	2.5 (3.4)	2.4 (3.3)	3.1 (3.8)	<0.001

Abbreviations: UKB = UK biobank, CKM= Cardiovascular-Kidney-Metabolic Syndrome, BMI= body mass index, SBP= systolic blood pressure, DBP= diastolic blood pressure, FPG= fasting plasma glucose, HbA1c= glycosylated hemoglobin, TG= total triglycerides, HDL-C= high-density lipoprotein cholesterol, LDL-C= low-density lipoprotein cholesterol, TC= total cholesterol, eGFR= estimated glomerular filtration rate.

Table S4 Baseline characteristics of participants for cross-sectional analyses in CHARLS

Characteristic	Total<br>N = 8550	Non advanced CKM<br>N = 6624	Advanced CKM<br>N = 1926	*P* value
Age	59.2 (9.6)	57.6 (9.1)	64.5 (9.5)	<0.001
Gender				<0.001
Female	4645 (54.3%)	3874 (58.5%)	771 (40.0%)	
Male	3905 (45.7%)	2750 (41.5%)	1155 (60.0%)	
Education				0.059
Above high school	794 (9.3%)	621 (9.4%)	173 (9.0%)	
High school	3427 (40.1%)	2610 (39.4%)	817 (42.4%)	
Less than high school	4329 (50.6%)	3393 (51.2%)	936 (48.6%)	
Drinking status				<0.001
Never	5050 (59.1%)	4026 (60.8%)	1024 (53.2%)	
Former/Current	3500 (40.9%)	2598 (39.2%)	902 (46.8%)	
Smoking status				<0.001
Never	5248 (61.4%)	4384 (66.2%)	864 (44.9%)	
Former/Current	3302 (38.6%)	2240 (33.8%)	1062 (55.1%)	
BMI (kg/m²)	24.0 (4.1)	23.9 (4.0)	24.1 (4.4)	<0.001
Waist (cm)	84.4 (12.4)	83.6 (12.1)	87.1 (13.2)	<0.001
Antidiabetic drugs				<0.001
No	7903 (92.4%)	6249 (94.3%)	1654 (85.9%)	
Yes	647 (7.6%)	375 (5.7%)	272 (14.1%)	
Lipid-lowering drugs				<0.001
No	8112 (94.9%)	6359 (96.0%)	1753 (91.0%)	
Yes	438 (5.1%)	265 (4.0%)	173 (9.0%)	
SBP (mmHg)	128.6 (16.3)	126.1 (15.8)	137.2 (17.1)	<0.001
DBP (mmHg)	74.3 (9.8)	73.5 (9.6)	77.2 (10.2)	<0.001
FPG (mg/dL)	109.9 (35.8)	107.4 (31.1)	118.5 (47.4)	<0.001
HbA1c (%)	5.3 (0.8)	5.2 (0.7)	5.5 (1.0)	<0.001
TG (mg/dL)	131.2 (108.5)	122.8 (88.8)	159.9 (155.1)	<0.001
HDL-C (mg/dL)	51.3 (15.3)	52.6 (15.2)	47.0 (14.6)	<0.001
LDL-C (mg/dL)	116.7 (33.2)	115.3 (32.6)	121.0 (35.8)	<0.001
TC (mg/dL)	194.2 (39.0)	192.5 (37.4)	200.0 (43.4)	<0.001
eGFR (mL/min/1.73m²)	92.3 (20.5)	99.6 (17.2)	66.8 (23.4)	<0.001
C-reactive protein (mg/L)	2.5 (5.1)	2.3 (4.8)	3.2 (6.0)	<0.001

Abbreviations: CHARLS= China Health and Retirement Longitudinal Study, CKM= Cardiovascular-Kidney-Metabolic Syndrome, BMI= body mass index, SBP= systolic blood pressure, DBP= diastolic blood pressure, FPG= fasting plasma glucose, HbA1c= glycosylated hemoglobin, TG= total triglycerides, HDL-C= high-density lipoprotein cholesterol, LDL-C= low-density lipoprotein cholesterol, TC= total cholesterol, eGFR= estimated glomerular filtration rate.

Table S5 Baseline characteristics of participants for longitudinal analyses in UK Biobank

Characteristic	Total<br>N = 186753	T1<br>N = 62251	T2<br>N = 62251	T3<br>N = 62251	*p* value
Age	55.9 (8.0)	54.3 (8.1)	56.5 (7.9)	56.8 (7.8)	<0.001
Gender					<0.001
Female	107332 (57.5%)	40149 (64.5%)	34382 (55.2%)	32801 (52.7%)	
Male	79421 (42.5%)	22102 (35.5%)	27869 (44.8%)	29450 (47.3%)	
Education					<0.001
Unkown	31262 (16.7%)	7242 (11.6%)	10662 (17.1%)	13358 (21.5%)	
College	60832 (32.6%)	24539 (39.4%)	19800 (31.8%)	16493 (26.5%)	
Other levels	94659 (50.7%)	30470 (48.9%)	31789 (51.1%)	32400 (52.0%)	
Drinking status					<0.001
Never	14348 (7.7%)	3989 (6.4%)	4542 (7.3%)	5817 (9.3%)	
Former/Current	172405 (92.3%)	58262 (93.6%)	57709 (92.7%)	56434 (90.7%)	
Smoking status					<0.001
Never	106305 (56.9%)	38275 (61.5%)	35614 (57.2%)	32416 (52.1%)	
Former/Current	80448 (43.1%)	23976 (38.5%)	26637 (42.8%)	29835 (47.9%)	
BMI (kg/m²)	27.3 (4.7)	24.7 (3.4)	27.2 (4.1)	29.9 (5.0)	<0.001
Waist (cm)	89.4 (13.2)	81.7 (10.7)	89.7 (11.6)	96.8 (12.6)	<0.001
Antidiabetic drugs					<0.001
No	178725 (95.7%)	61066 (98.1%)	59563 (95.7%)	58013 (93.2%)	
Yes	8028 (4.3%)	1185 (1.9%)	2688 (4.3%)	4238 (6.8%)	
Lipid-lowering drugs					<0.001
No	150150 (80.4%)	52251 (83.9%)	49801 (80.0%)	48071 (77.2%)	
Yes	36603 (19.6%)	10000 (16.1%)	12450 (20.0%)	14180 (22.8%)	
SBP (mmHg)	119.7 (14.4)	115.2 (13.1)	119.5 (14.2)	124.8 (15.3)	<0.001
DBP (mmHg)	69.4 (9.2)	67.1 (8.5)	69.4 (9.0)	72.0 (9.6)	<0.001
FPG (mg/dL)	91.0 (16.1)	86.7 (10.6)	89.8 (12.8)	96.4 (21.2)	<0.001
HbA1c (%)	5.4 (0.6)	5.3 (0.4)	5.4 (0.4)	5.6 (0.7)	<0.001
TG (mg/dL)	150.5 (83.4)	88.0 (27.6)	137.6 (44.7)	225.9 (91.3)	<0.001
HDL-C (mg/dL)	56.8 (14.3)	63.9 (14.3)	56.7 (13.2)	49.9 (11.7)	<0.001
LDL-C (mg/dL)	94.1 (31.4)	93.5 (30.2)	94.3 (31.3)	94.8 (33.0)	<0.001
TC (mg/dL)	181.1 (35.7)	178.3 (33.0)	183.0 (35.4)	182.1 (38.2)	<0.001
eGFR (mL/min/1.73m²)	102.4 (16.4)	105.8 (14.2)	102.5 (16.1)	98.6 (18.3)	<0.001
C-reactive protein (mg/L)	2.4 (3.3)	0.7 (0.6)	1.9 (1.8)	4.6 (4.6)	<0.001
CTI	8.8 (0.8)	8.0 (0.4)	8.8 (0.2)	9.7 (0.4)	<0.001

Abbreviations: UKB = UK biobank, CKM= Cardiovascular-Kidney-Metabolic Syndrome, BMI= body mass index, SBP= systolic blood pressure, DBP= diastolic blood pressure, FPG= fasting plasma glucose, HbA1c= glycosylated hemoglobin, TG= total triglycerides, HDL-C= high-density lipoprotein cholesterol, LDL-C= low-density lipoprotein cholesterol, TC= total cholesterol, eGFR= estimated glomerular filtration rate.

Table S6 Baseline characteristics of participants for longitudinal analyses in CHARLS

Characteristic	Total<br>N = 3673	T1<br>N = 1225	T2<br>N = 1224	T3<br>N = 1224	*p* value
Age	61.2 (8.4)	61.3 (8.7)	61.2 (8.3)	61.0 (8.4)	0.612
Gender					<0.001
Female	2211 (60.2%)	639 (52.2%)	742 (60.6%)	830 (67.8%)	
Male	1462 (39.8%)	586 (47.8%)	482 (39.4%)	394 (32.2%)	
Education					0.733
Above high school	317 (8.6%)	110 (9.0%)	107 (8.7%)	100 (8.2%)	
High school	1472 (40.1%)	486 (39.7%)	488 (39.9%)	498 (40.7%)	
Less than high school	1884 (51.3%)	629 (51.3%)	629 (51.4%)	626 (51.1%)	
Drinking status					<0.001
Never	2081 (56.7%)	632 (51.6%)	684 (55.9%)	765 (62.5%)	
Former/Current	1592 (43.3%)	593 (48.4%)	540 (44.1%)	459 (37.5%)	
Smoking status					<0.001
Never	2333 (63.5%)	707 (57.7%)	790 (64.5%)	836 (68.3%)	
Former/Current	1340 (36.5%)	518 (42.3%)	434 (35.5%)	388 (31.7%)	
BMI (kg/m²)	24.3 (4.9)	22.6 (4.3)	24.2 (4.7)	26.0 (5.1)	<0.001
Waist (cm)	84.4 (13.8)	79.5 (12.9)	84.2 (14.5)	89.4 (12.0)	<0.001
Antidiabetic drugs					<0.001
No	3449 (93.9%)	1203 (98.2%)	1148 (93.8%)	1098 (89.7%)	
Yes	224 (6.1%)	22 (1.8%)	76 (6.2%)	126 (10.3%)	
Lipid-lowering drugs					<0.001
No	3511 (95.6%)	1200 (97.9%)	1174 (95.9%)	1137 (93.0%)	
Yes	162 (4.4%)	25 (2.1%)	50 (4.1%)	87 (7.0%)	
SBP (mmHg)	126.6 (16.7)	120.9 (15.1)	126.2 (16.3)	131.5 (17.6)	<0.001
DBP (mmHg)	73.9 (10.4)	69.7 (9.2)	73.7 (10.1)	77.6 (11.0)	<0.001
FPG (mg/dL)	99.7 (29.4)	90.2 (11.9)	96.0 (16.6)	112.9 (43.5)	<0.001
HbA1c (%)	6.0 (1.0)	5.7 (0.5)	5.9 (0.7)	6.3 (1.4)	<0.001
TG (mg/dL)	134.2 (82.6)	77.7 (21.7)	115.8 (32.2)	209.3 (99.1)	<0.001
HDL-C (mg/dL)	52.4 (11.9)	56.9 (12.7)	52.5 (11.2)	47.8 (9.8)	<0.001
LDL-C (mg/dL)	106.0 (33.5)	101.8 (32.1)	109.4 (33.2)	106.7 (34.8)	<0.001
TC (mg/dL)	185.2 (36.5)	174.2 (30.3)	185.1 (33.6)	196.4 (41.3)	<0.001
eGFR (mL/min/1.73m²)	99.0 (18.5)	103.0 (16.8)	98.8 (17.9)	96.1 (19.4)	<0.001
C-reactive protein (mg/L)	2.3 (3.4)	0.8 (0.7)	1.8 (1.8)	4.4 (4.9)	<0.001
CTI	8.8 (0.8)	7.9 (0.4)	8.7 (0.2)	9.7 (0.5)	<0.001

Abbreviations: CHARLS= China Health and Retirement Longitudinal Study, CKM= Cardiovascular-Kidney-Metabolic Syndrome, BMI= body mass index, SBP= systolic blood pressure, DBP= diastolic blood pressure, FPG= fasting plasma glucose, HbA1c= glycosylated hemoglobin, TG= total triglycerides, HDL-C= high-density lipoprotein cholesterol, LDL-C= low-density lipoprotein cholesterol, TC= total cholesterol, eGFR= estimated glomerular filtration rate.

Table S7 Baseline characteristics comparison between included and excluded participants for longitudinal analysis (UKB and CHARLS)

Characteristic	UK Biobank			CHARLS		
	Included N=186,753	Excluded N=36,962	*P* value	Included N=3,673	Excluded N=3,138	*P* value
Age, mean (SD)	55.9 (8.0)	56.4 (8.3)	0.206	61.2 (8.4)	61.9 (8.7)	0.284
Gender, n (%)			0.259			0.327
Female	107,332 (57.5%)	20,994 (56.8%)		2,211 (60.2%)	1,839 (58.6%)	
Male	79,421 (42.5%)	15,968 (43.2%)		1,462 (39.8%)	1,299 (41.4%)	
BMI (kg/m²), mean (SD)	27.3 (4.7)	27.7 (5.0)	0.173	24.3 (14.3)	24.8 (14.7)	0.305
Waist (cm), mean (SD)	89.4 (13.2)	90.2 (13.6)	0.228	84.4 (13.8)	85.1 (14.2)	0.336
CTI, mean (SD)	8.8 (0.8)	8.9 (0.9)	0.364	8.8 (0.8)	8.9 (0.9)	0.392

Abbreviations: UKB=UK biobank, CHARLS= China Health and Retirement Longitudinal Study, CTI= C-reactive protein to triglyceride glucose index, BMI= body mass index.

Table S8 Association of CTI with risk of advanced CKM based on cross-sectional data after excluding participants with cancer

	Model 1		Model 2		Model 3	
	OR(95%CI)	*P* value	OR(95%CI)	*P* value	OR(95%CI)	*P* value
NHANES						
CTI continuous	1.61 (1.46—1.79)	< 0.001	1.41 (1.30—1.56)	< 0.001	1.38 (1.27—1.39)	< 0.001
T1	Ref	Ref	Ref	Ref	Ref	Ref
T2	1.83 (1.66—2.08)	< 0.001	1.20 (1.09—1.38)	< 0.001	1.18 (1.05—1.37)	0.008
T3	2.71 (2.36—3.16)	< 0.001	1.76 (1.65—2.06)	< 0.001	1.72 (1.53—1.96)	< 0.001
P for trend		< 0.001		< 0.001		< 0.001
UKB						
CTI continuous	1.81 (1.71—1.93)	< 0.001	1.63 (1.53—1.74)	< 0.001	1.54 (1.47—1.66)	< 0.001
T1	Ref	Ref	Ref	Ref	Ref	Ref
T2	1.86 (1.76—1.97)	< 0.001	1.38 (1.29—1.51)	< 0.001	1.31 (1.23—1.42)	< 0.001
T3	3.13 (2.94—3.39)	< 0.001	2.32 (2.15—2.53)	< 0.001	2.13 (1.98—2.35)	< 0.001
P for trend		< 0.001		< 0.001		< 0.001
CHARLS						
CTI continuous	1.88 (1.65—2.16)	< 0.001	1.75 (1.61—1.95)	< 0.001	1.46 (1.31—1.64)	< 0.001
T1	Ref	Ref	Ref	Ref	Ref	Ref
T2	1.56 (1.41—1.81)	< 0.001	1.53 (1.31—1.71)	< 0.001	1.42 (1.21—1.68)	< 0.001
T3	3.03 (2.78—3.39)	< 0.001	2.82 (2.51—3.16)	< 0.001	2.39 (2.13—2.71)	< 0.001
P for trend		< 0.001		< 0.001		< 0.001

Model 1: crude unadjusted model.

Model 2: Adjusted for age and gender

Model 3: Further adjusted for smoking status, drinking status, BMI, SBP, DBP, LDL-C, eGFR, antidiabetic drugs, lipid-lowering drugs, and baseline CKM stage based on Model 2.

Abbreviations: NHANES= National Health and Nutrition Examination Survey, UKB=UK biobank, CHARLS= China Health and Retirement Longitudinal Study, CTI=C-reactive protein to triglyceride glucose index. CKM= cardiovascular-kidney–metabolic syndrome, BMI= body mass index, SBP= systolic blood pressure, DBP= diastolic blood pressure, LDL-C= Low-density lipoprotein cholesterol, eGFR= estimated glomerular filtration rate, HR= hazard ratio, CI= confidence interval.

Table S9 Association of CTI with risk of new-onset advanced CKM based on longitudinal data after excluding participants with cancer

		Model 1		Model 2		Model 3	
	Events/n	HR (95%CI)	*P* value	HR (95%CI)	*P* value	HR (95%CI)	*P* value
UKB							
CTI continuous	22,938 /173,813	1.37 (1.24—1.53)	< 0.001	1.27 (1.14—1.43)	< 0.001	1.23 (1.15—1.32)	< 0.001
T1	6,431/57,937	Ref	Ref	Ref	Ref	Ref	Ref
T2	7,784/57,938	1.34 (1.40—1.51)	< 0.001	1.17 (1.16—1.28)	< 0.001	1.13 (1.06—1.23)	< 0.001
T3	8,723/57,938	1.82 (1.71—1.95)	< 0.001	1.49 (1.39—1.62)	< 0.001	1.44 (1.34—1.55)	< 0.001
P for trend			< 0.001		< 0.001		< 0.001
CHARLS							
CTI continuous	319/3557	1.41 (1.19—1.65)	< 0.001	1.24 (1.06—1.47)	0.004	1.19 (1.03—1.36)	0.031
T1	82/1186	Ref	Ref	Ref	Ref	Ref	Ref
T2	107/1186	1.49 (1.18—1.83)	0.007	1.32 (1.08—1.56)	< 0.001	1.26 (1.05—1.52)	0.039
T3	130/1185	1.86 (1.52—2.23)	< 0.001	1.59 (1.29—1.93)	< 0.001	1.45 (1.23—1.71)	< 0.001
P for trend			< 0.001		< 0.001		< 0.001

Model 1: crude unadjusted model.

Model 2: Adjusted for age and gender

Model 3: Further adjusted for smoking status, drinking status, BMI, SBP, DBP, LDL-C, eGFR, antidiabetic drugs, lipid-lowering drugs, and baseline CKM stage based on Model 2.

Abbreviations: NHANES= National Health and Nutrition Examination Survey, UKB=UK biobank, CHARLS= China Health and Retirement Longitudinal Study, CTI=C-reactive protein to triglyceride glucose index. CKM= cardiovascular-kidney–metabolic syndrome, BMI= body mass index, SBP= systolic blood pressure, DBP= diastolic blood pressure, LDL-C= Low-density lipoprotein cholesterol, eGFR= estimated glomerular filtration rate, HR= hazard ratio, CI= confidence interval.

Table S10 Association of CTI with risk of advanced CKM based on cross-sectional data from NHANES, UKB, and CHARLS after excluding participants with acute infection, severe autoimmune diseases, or recent major surgery

	Model 1		Model 2		Model 3	
	OR(95%CI)	*P* value	OR(95%CI)	*P* value	OR(95%CI)	*P* value
NHANES						
CTI continuous	1.59 (1.31–1.85)	< 0.001	1.38 (1.16–1.62)	< 0.001	1.29 (1.12–1.48)	< 0.001
T1	Ref		Ref		Ref	
T2	1.77 (1.46–2.09)	< 0.001	1.18 (1.02–1.44)	0.029	1.14 (1.01–1.38)	0.045
T3	2.68 (2.25–3.15)	< 0.001	1.65 (1.36–2.01)	< 0.001	1.59 (1.38–1.83)	< 0.001
P for trend		< 0.001		< 0.001		< 0.001
UKB						
CTI continuous	1.68 (1.51–1.86)	< 0.001	1.59 (1.47–1.74)	< 0.001	1.47 (1.35–1.61)	< 0.001
T1	Ref		Ref		Ref	
T2	1.73 (1.56–1.95)	< 0.001	1.29 (1.22–1.46)	< 0.001	1.23 (1.12–1.36)	< 0.001
T3	2.95 (2.74–3.18)	< 0.001	2.12 (1.93–2.31)	< 0.001	1.94 (1.79–2.09)	< 0.001
P for trend		< 0.001		< 0.001		< 0.001
CHARLS						
CTI continuous	1.88 (1.60–2.23)	< 0.001	1.74 (1.50–2.04)	< 0.001	1.46 (1.25–1.72)	< 0.001
T1	Ref		Ref		Ref	
T2	1.61 (1.39–1.88)	< 0.001	1.52 (1.31–1.78)	< 0.001	1.43 (1.25–1.64)	< 0.001
T3	3.05 (2.68–3.49)	< 0.001	2.79 (2.44–3.22)	< 0.001	2.45 (2.18–2.73)	< 0.001
P for trend		< 0.001		< 0.001		< 0.001

Model 1: crude unadjusted model.

Model 2: Adjusted for age and gender

Model 3: Further adjusted for smoking status, drinking status, BMI, SBP, DBP, LDL-C, eGFR, antidiabetic drugs, lipid-lowering drugs, and baseline CKM stage based on Model 2.

Abbreviations: NHANES= National Health and Nutrition Examination Survey, UKB=UK biobank, CHARLS= China Health and Retirement Longitudinal Study, CTI=C-reactive protein to triglyceride glucose index. CKM= cardiovascular-kidney–metabolic syndrome, BMI= body mass index, SBP= systolic blood pressure, DBP= diastolic blood pressure, LDL-C= Low-density lipoprotein cholesterol, eGFR= estimated glomerular filtration rate, HR= hazard ratio, CI= confidence interval.

Table S11 Association of CTI with risk of new-onset advanced CKM based on longitudinal data in UKB and CHARLS after excluding participants with acute infection, severe autoimmune diseases, or recent major surgery

		Model 1		Model 2		Model 3	
	Events/n	HR (95%CI)	*P* value	HR (95%CI)	*P* value	HR (95%CI)	*P* value
UKB							
CTI continuous	24,277 / 181,154	1.39 (1.26–1.53)	< 0.001	1.28 (1.21–1.41)	< 0.001	1.24 (1.15–1.35)	< 0.001
T1	5,518 / 60,384	Ref		Ref		Ref	
T2	7,926 / 60,384	1.43 (1.31–1.64)	< 0.001	1.23 (1.12–1.35)	< 0.001	1.18 (1.06–1.34)	0.025
T3	10,833 / 60,386	1.82 (1.68–2.07)	< 0.001	1.51 (1.36–1.67)	< 0.001	1.45 (1.32–1.51)	< 0.001
P for trend			< 0.001		< 0.001		< 0.001
CHARLS							
CTI continuous	294 / 3,463	1.42 (1.19–1.63)	< 0.001	1.24 (1.07–1.44)	< 0.001	1.19 (1.02–1.38)	0.033
T1	81 / 1,154	Ref		Ref		Ref	
T2	104 / 1,154	1.49 (1.25–1.76)	0.003	1.33 (1.15–1.62)	< 0.001	1.29 (1.10–1.55)	< 0.001
T3	127 / 1,155	1.78 (1.51–2.06)	< 0.001	1.57 (1.25–1.94)	< 0.001	1.52 (1.30–1.79)	< 0.001
P for trend			< 0.001		< 0.001		< 0.001

Model 1: crude unadjusted model.

Model 2: Adjusted for age and gender

Model 3: Further adjusted for smoking status, drinking status, BMI, SBP, DBP, LDL-C, eGFR, antidiabetic drugs, lipid-lowering drugs, and baseline CKM stage based on Model 2.

Abbreviations: NHANES= National Health and Nutrition Examination Survey, UKB=UK biobank, CHARLS= China Health and Retirement Longitudinal Study, CTI=C-reactive protein to triglyceride glucose index. CKM= cardiovascular-kidney–metabolic syndrome, BMI= body mass index, SBP= systolic blood pressure, DBP= diastolic blood pressure, LDL-C= Low-density lipoprotein cholesterol, eGFR= estimated glomerular filtration rate, HR= hazard ratio, CI= confidence interval.

Table S12 Association of CTI with risk of advanced CKM based on cross-sectional data from NHANES, UKB, and CHARLS using multiple imputation data

	Model 1		Model 2		Model 3	
	OR(95%CI)	*P* value	OR(95%CI)	*P* value	OR(95%CI)	*P* value
NHANES						
CTI continuous	1.64 (1.48–1.79)	< 0.001	1.49 (1.32–1.68)	< 0.001	1.33 (1.16–1.52)	< 0.001
T1	Ref		Ref		Ref	
T2	1.94 (1.61–2.25)	< 0.001	1.23 (1.04–1.51)	0.018	1.19 (1.02–1.46)	0.039
T3	2.86 (2.35–3.34)	< 0.001	1.85 (1.48–2.23)	< 0.001	1.67 (1.36–1.97)	< 0.001
P for trend		< 0.001		< 0.001		< 0.001
UKB						
CTI continuous	1.92 (1.75–2.04)	< 0.001	1.68 (1.60–1.78)	< 0.001	1.57 (1.45–1.71)	< 0.001
T1	Ref		Ref		Ref	
T2	1.88 (1.71–2.09)	< 0.001	1.43 (1.29–1.55)	< 0.001	1.26 (1.15–1.38)	< 0.001
T3	3.19 (2.90–3.53)	< 0.001	2.35 (2.15–2.59)	< 0.001	2.04 (1.85–2.26)	< 0.001
P for trend		< 0.001		< 0.001		< 0.001
CHARLS						
CTI continuous	1.91 (1.69–2.24)	< 0.001	1.82 (1.63–2.09)	< 0.001	1.45 (1.27–1.66)	< 0.001
T1	Ref		Ref		Ref	
T2	1.68 (1.44–2.03)	< 0.001	1.56 (1.33–1.82)	< 0.001	1.39 (1.21–1.64)	< 0.001
T3	3.13 (2.68–3.58)	< 0.001	2.91 (2.50–3.33)	< 0.001	2.26 (1.98–2.61)	< 0.001
P for trend		< 0.001		< 0.001		< 0.001

Model 1: crude unadjusted model.

Model 2: Adjusted for age and gender

Model 3: Further adjusted for smoking status, drinking status, BMI, SBP, DBP, LDL-C, eGFR, antidiabetic drugs, lipid-lowering drugs, and baseline CKM stage based on Model 2.

Abbreviations: NHANES= National Health and Nutrition Examination Survey, UKB=UK biobank, CHARLS= China Health and Retirement Longitudinal Study, CTI=C-reactive protein to triglyceride glucose index. CKM= cardiovascular-kidney–metabolic syndrome, BMI= body mass index, SBP= systolic blood pressure, DBP= diastolic blood pressure, LDL-C= Low-density lipoprotein cholesterol, eGFR= estimated glomerular filtration rate, HR= hazard ratio, CI= confidence interval.

Table S13 Association of CTI with risk of new-onset advanced CKM based on longitudinal data in UKB and CHARLS using multiple imputation data

		Model 1		Model 2		Model 3	
	Events/n	HR (95%CI)	*P* value	HR (95%CI)	*P* value	HR (95%CI)	*P* value
UKB							
CTI continuous	33,538 / 223,715	1.47 (1.35–1.63)	< 0.001	1.35 (1.29–1.43)	< 0.001	1.23 (1.16–1.32)	< 0.001
T1	6,711 / 74,571	Ref		Ref		Ref	
T2	11,185 / 74,572	1.42 (1.30–1.53)	< 0.001	1.22 (1.16–1.28)	< 0.001	1.18 (1.12–1.30)	< 0.001
T3	15,642 / 74,572	1.93 (1.79–2.06)	< 0.001	1.59 (1.51–1.72)	< 0.001	1.42 (1.33–1.54)	< 0.001
P for trend			< 0.001		< 0.001		< 0.001
CHARLS							
CTI continuous	604 / 6,811	1.44 (1.14–1.73)	< 0.001	1.31 (1.10–1.59)	< 0.001	1.24 (1.04–1.50)	0.027
T1	181 / 2,270	Ref		Ref		Ref	
T2	202 / 2,271	1.59 (1.31–1.83)	< 0.001	1.39 (1.10–1.65)	0.007	1.30 (1.08–1.58)	0.015
T3	221 / 2,270	1.96 (1.60–2.37)	< 0.001	1.69 (1.31–2.01)	< 0.001	1.48 (1.23–1.79)	< 0.001
P for trend			< 0.001		< 0.001		< 0.001

Model 1: crude unadjusted model.

Model 2: Adjusted for age and gender

Model 3: Further adjusted for smoking status, drinking status, BMI, SBP, DBP, LDL-C, eGFR, antidiabetic drugs, lipid-lowering drugs, and baseline CKM stage based on Model 2.

Abbreviations: NHANES= National Health and Nutrition Examination Survey, UKB=UK biobank, CHARLS= China Health and Retirement Longitudinal Study, CTI= C-reactive protein to triglyceride glucose index. CKM= cardiovascular-kidney–metabolic syndrome, BMI= body mass index, SBP= systolic blood pressure, DBP= diastolic blood pressure, LDL-C= Low-density lipoprotein cholesterol, eGFR= estimated glomerular filtration rate, HR= hazard ratio, CI= confidence interval.

Table S14 Association of CTI with risk of new-onset advanced CKM based on longitudinal data in UKB and CHARLS using the competing risk model

		Model 1		Model 2		Model 3	
	Events/n	sHR (95%CI)	*P* value	sHR (95%CI)	*P* value	sHR (95%CI)	*P* value
UKB							
CTI continuous	26,534 / 186,753	1.37 (1.21–1.58)	< 0.001	1.28 (1.19–1.43)	< 0.001	1.17 (1.08–1.30)	< 0.001
T1	6,263 / 62,251	Ref		Ref		Ref	
T2	8,856 / 62,251	1.34 (1.22–1.52)	< 0.001	1.15 (1.06–1.27)	0.018	1.13 (1.02–1.24)	0.026
T3	11,415 / 62,251	1.78 (1.62–2.01)	< 0.001	1.48 (1.35–1.66)	< 0.001	1.32 (1.19–1.49)	< 0.001
P for trend			< 0.001		< 0.001		< 0.001
CHARLS							
CTI continuous	345 / 3,673	1.40 (1.15–1.75)	< 0.001	1.25 (1.04–1.57)	< 0.001	1.16 (1.01–1.35)	0.033
T1	91 / 1,225	Ref		Ref		Ref	
T2	110 / 1,224	1.48 (1.25–1.81)	< 0.001	1.32 (1.10–1.66)	0.005	1.23 (1.03–1.51)	0.014
T3	144 / 1,224	1.86 (1.49–2.35)	< 0.001	1.55 (1.21–1.95)	< 0.001	1.37 (1.13–1.71)	< 0.001
P for trend			< 0.001		< 0.001		< 0.001

Model 1: crude unadjusted model.

Model 2: Adjusted for age and gender

Model 3: Further adjusted for smoking status, drinking status, BMI, SBP, DBP, LDL-C, eGFR, antidiabetic drugs, lipid-lowering drugs, and baseline CKM stage based on Model 2.

Abbreviations: NHANES= National Health and Nutrition Examination Survey, UKB=UK biobank, CHARLS= China Health and Retirement Longitudinal Study, CTI=C-reactive protein to triglyceride glucose index. CKM= cardiovascular-kidney–metabolic syndrome, BMI= body mass index, SBP= systolic blood pressure, DBP= diastolic blood pressure, LDL-C= Low-density lipoprotein cholesterol, eGFR= estimated glomerular filtration rate, sHR= subdistribution hazard ratio, CI= confidence interval.
